# Enhancement of the ionoacoustic effect through ultrasound and photoacoustic contrast agents

**DOI:** 10.1038/s41598-021-81964-4

**Published:** 2021-02-01

**Authors:** Julie Lascaud, Pratik Dash, Matthias Würl, Hans-Peter Wieser, Benjamin Wollant, Ronaldo Kalunga, Walter Assmann, Dirk-André Clevert, Alfredo Ferrari, Paola Sala, Alessandro Stuart Savoia, Katia Parodi

**Affiliations:** 1 Department for Medical Physics, Ludwig-Maximilians-Universität München (LMU Munich), 85748 Garching b. München, Germany; 2 Interdisciplinary Ultrasound-Center, Department of Radiology, University of Munich-Grosshadern Campus, Munich, Germany; 3 Universitätsklinikum, Heidelberg, Germany; 4 Gangneu-Wonju National University, Gangneung, Wonju, South Korea; 5 Italian National Institute for Nuclear Physics (INFN), 20133 Milan, Italy; 6 Department of Engineering, Roma Tre University, 00146 Rome, Italy

**Keywords:** Medical imaging, Radiotherapy, Acoustics, Photoacoustics

## Abstract

The characteristic depth dose deposition of ion beams, with a maximum at the end of their range (Bragg peak) allows for local treatment delivery, resulting in better sparing of the adjacent healthy tissues compared to other forms of external beam radiotherapy treatments. However, the optimal clinical exploitation of the favorable ion beam ballistic is hampered by uncertainties in the in vivo Bragg peak position. Ionoacoustics is based on the detection of thermoacoustic pressure waves induced by a properly pulsed ion beam (e.g., produced by modern compact accelerators) to image the irradiated volume. Co-registration between ionoacoustics and ultrasound imaging offers a promising opportunity to monitor the ion beam and patient anatomy during the treatment. Nevertheless, the detection of the ionoacoustic waves is challenging due to very low pressure amplitudes and frequencies (mPa/kHz) observed in clinical applications. We investigate contrast agents to enhance the acoustic emission. Ultrasound microbubbles are used to increase the ionoacoustic frequency around the microbubble resonance frequency. Moreover, India ink is investigated as a possible mean to enhance the signal amplitude by taking advantage of additional optical photon absorption along the ion beam and subsequent photoacoustic effect. We report amplitude increase of up to 200% of the ionoacoustic signal emission in the MHz frequency range by combining microbubbles and India ink contrast agents.

## Introduction

Ionoacoustics relies on the detection of thermoacoustic waves generated when charged particles from pulsed ion beams stop in matter^1^. The deceleration of ions penetrating a medium results in energy deposition per unit of mass, i.e., dose which generates pressure waves if the amount of energy is delivered over a short period of time. In stress-confinement regime, i.e., when the duration of the ion beam pulse is shorter than the time required for the acoustic wave to travel across the heated volume, the evolution of the pressure wave over time describes the dose variation along the propagation axis^2^. Therefore, this distinctive feature can be used to monitor the dose distribution which is of particular interest in ion beam therapy. In the following this will be discussed in more detail in the context of proton beam therapy. Unlike photons, protons offer a characteristic inverse depth dose profile with a pronounced maximum at the end of their range, the so-called Bragg peak. The direct benefit of proton beams in external beam radiotherapy is a better sparing of the healthy tissue surrounding the tumor due to the localized dose deposition inside the tumor volume. Herewith, negative treatment side effects and risk of secondary cancer development are reduced, but full clinical exploitation of the ballistic advantages for improved treatment outcome requires a precise knowledge of the in vivo Bragg peak position. Nowadays, the targeted dose obtained from the treatment planning system is affected by uncertainties in the knowledge of the patient position at the actual day of treatment, possible anatomical changes within and between treatment fractions and inaccuracies in the derivation of stopping power ratios of protons in tissue relative to water from the underlying X-ray computed tomography images, reflecting the attenuation of photons^3–5^. Although range verification techniques based on the detection of proton-induced secondary photon emissions are being well-established and reached the stage of clinical translation^6,7^, they rely on nuclear-based processes and thus only offer indirect information of the dose distribution. Moreover, without supplemental in situ imaging devices, such techniques are not providing any in vivo information on the patient anatomy. Therefore, it is not possible to confirm with certainty whether the Bragg peak position is inside the tumor volume or in an adjacent organ at risk. In contrast, ionoacoustics is based on the detection of the direct acoustic signal resulting from the energy deposition which is particularly enhanced in the Bragg peak region. Therefore, ionoacoustic signals encode all the information on the dose, allowing either to determine the proton beam range from time-of-flight measurements^8^ or to reconstruct the full-dose distribution^9^. It is worth noting that the dose dependence of the ionoacoustic signals also implies change of the pressure wave frequency and amplitude within the treatment field as the dose distribution could be distorted due to heterogeneous tissue composition or variation in the proton beam energy (adapted during the treatment to reach a specific penetration depth). As ionoacoustics is based on ultrasonic wave detection, it can readily be combined with ultrasonic images of the anatomy providing a simultaneous monitoring of the proton beam and patient anatomy.

However, the low pressure amplitudes and the low frequency range, in the order of some mPa in the 10 to 50 kHz range for clinically relevant proton beam energies^10^, make the detection of ionoacoustic signals challenging and compromise the co-integration with ultrasound imaging systems which operate in the MHz range. Increasing the detection sensitivity is difficult, which remains the main challenge to apply ionoacoustics for in vivo online range monitoring in proton therapy. Therefore, any enhancement of the ionoacoustic signals would facilitate the integration of ionoacoustics for pre-clinical and clinical applications. In this context, spherical gold markers were recently proposed^11^. Exploiting the large difference in acoustic impedance between the metal and tissue, the irradiated marker acts as an acoustic resonator, resulting in frequency-modulated ionoacoustic pulses for which the modulation frequency depends on the marker dimension and can reach the MHz frequency range for millimeter-sized spheres. However, the method is limited to proton beams passing through the millimeter-sized maker whereas pencil beams are usually moved in the centimeter scale during the treatment to cover the full tumor volume. Furthermore, implanting a marker is invasive and the metallic object could distort the dose or cause artifacts in the pre-treatment imaging.

Hereby, we investigate for the first time the use of contrast agents (CA) to either enhance the signal amplitude or its frequency. The ionoacoustic signals generated in SonoVue microbubbles, India ink and a mixture India ink/microbubbles are assessed for a 22 MeV pulsed proton beam. Gas-filled microbubbles are well-established in ultrasound imaging^12–14^. The microbubble response to an incident pressure field (expansion and contraction of the bubble volume) leads to additional scattering of the acoustic waves and harmonic generation, for incident peak negative pressure inferior to the MPa level in the MHz frequency range. Injected intravenously, the insonicated microbubbles increase the contrast of ultrasound images. The micrometric size of the bubbles (with diameter from ${2}\,{\upmu \hbox {m}}$ to ${10}\,{\upmu \hbox {m}}$) induces resonance frequencies in the MHz range^15^, particularly adapted to ultrasound imaging. It has been recently shown that radiation sensitive polymer-based nanodroplets vaporize during proton irradiation, forming a microbubble cloud in regions proximal to the Bragg peak^16^ which can be imaged by ultrasound at the end of the treatment. Here, we hypothesized that microbubbles could increase the ionoacoustic signal frequency and amplitude. This could be caused by different phenomena. First, due to direct interactions either with the proton beam or secondary emissions which could damage the polymer shell of the SonoVue microbubbles, resulting in microbubble cavitation and subsequent high pressure and broadband acoustic emission. Alternatively, microbubble oscillations triggered by the ionoacoustic pressure could result in microbubble resonance at the proton pulse frequency and its harmonics. Furthermore, it has been reported that proton beams in water induce luminescence through the production of optical photons^17^. At pre-clinical and clinical proton beam energies, these photons mainly result from radicals produced in water along the irradiated path and the number of photons generated is proportional to the deposited dose^18,19^. Hence, the optical photon distribution follows the dose distribution and the energy deposition resulting from their absorption could contribute to the ionoacoustic signal. As a consequence, increasing the absorption of the optical photons could increase the ionoacoustic pressure due to an additional photoacoustic effect. In this work we investigate the ionoacoustic response obtained from these two types of CA (microbubbles and India ink-based) depending on the time structure of the pulsed beam (temporal excitation) and the dose distribution (spatial excitation) for low-energy mono-energetic proton beams.

## Results

### Contrast agents and experimental setup

**Figure 1:**
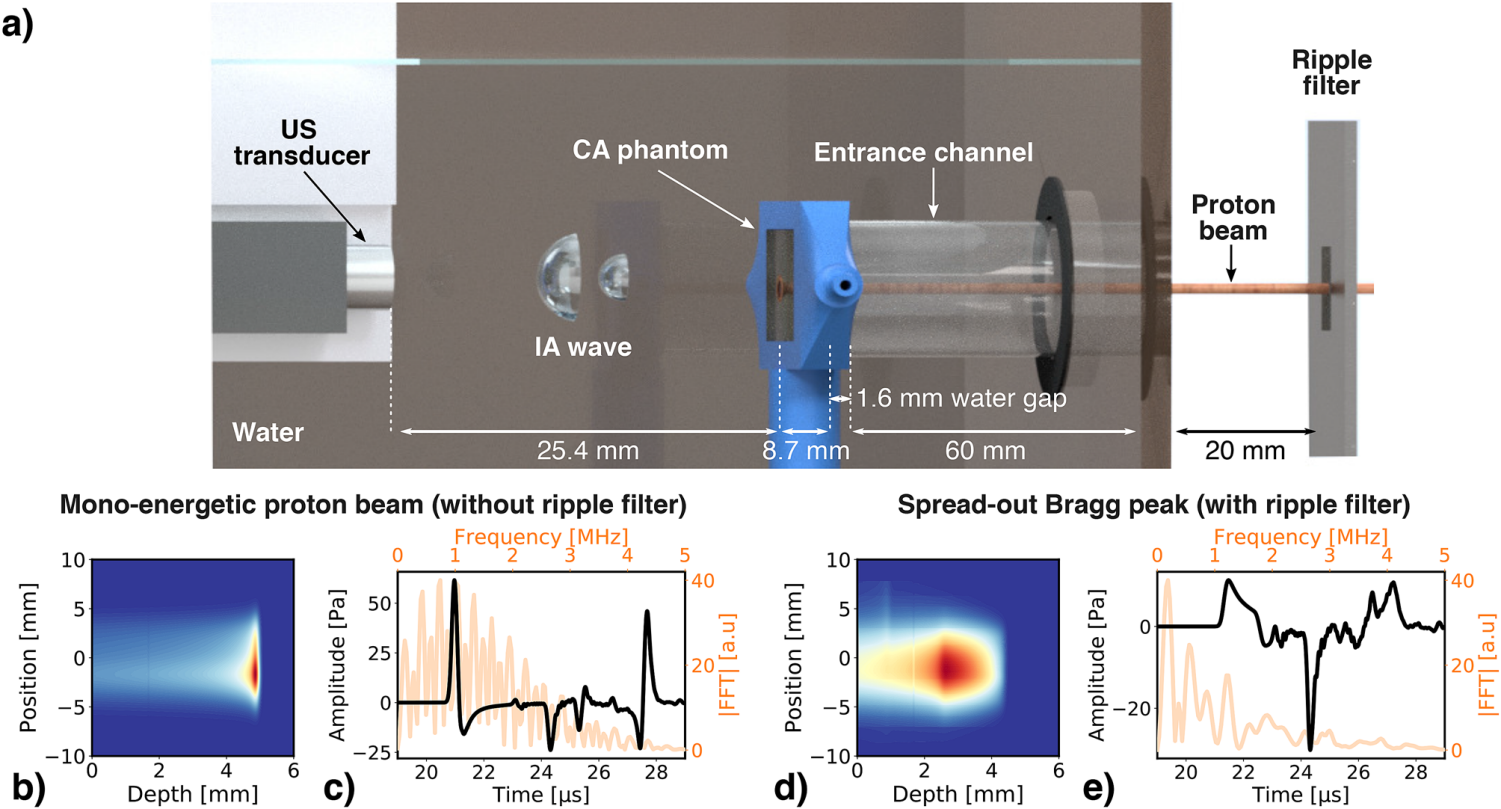
Experimental setup of the ionoacoustic CA study. (**a**) Schematic of the experimental setup in the case of irradiation with the ripple filter. The proton beam enters the water tank through a ${50}\,{\upmu \hbox {m}}$-thick polyimide foil and stops inside the CA phantom. The ionoacoustic (IA) pressure waves are measured by a CMUT single-element on the proton beam axis. (**b**) Laterally integrated 2D dose map of the 22 MeV proton beam in water, with a range of 4.92 mm (corresponding to 80 % of dose fall-off). (**c**) Simulated ionoacoustic emission resulting from the mono-energetic 22 MeV proton beam for a single proton pulse of 200 ns in water in time (black) and frequency-domain (orange). (**d**) Laterally integrated 2D dose map of the spread-out 22  MeV proton beam in water, with a range of 3.04 mm (corresponding to 90 % of dose fall-off). (**e**) Simulated ionoacoustic emission resulting for the spread-out proton beam from a single proton pulse of 200 ns in water in time (black) and frequency-domain (orange). The dose maps, obtained from Fluka2021.1beta, are the averaged values over 6 randomly sampled pencil beams.

The experiments were performed at the Tandem accelerator of the Maier-Leibnitz-Laboratory in Munich (Germany) with a 22 MeV pulsed proton beam irradiating a water tank. Figure 1a shows the typical setup used for the experiments. The proton beam enters the water tank through an air entrance channel terminated by a ${50}\,{\upmu \hbox {m}}$-thick polyimide foil. The beam stops inside a phantom also sealed by polyimide foils on both sides, used to contain the CA and placed inside the water tank. The details of the setup, including the polyimide foil positions, can be seen in Fig. S1 of the supporting information. The ultrasonic transducer, a 12 MHz CMUT (Capacitive Micromachined Ultrasonic Transducer) single-element from ACULAB (Acoustoelectronics Laboratory of the Università degli Studi Roma Tre), was immersed in water and aligned with the CA phantom and the water tank entrance channel, along the proton beam axis. During the experiments, the CA phantom was consecutively filled with three different CA: SonoVue microbubbles diluted in deionized water (MB), a mixture of SonoVue microbubbles and India ink diluted in deionized water (MBink) and India ink diluted in deionized water (India ink).

In water, the 22 MeV mono-energetic proton beam leads to a sharp dose deposition ($FWHM_{axial} = {430}\,{\upmu \hbox {m}}$, see Fig. 1b). As illustrated in Fig. 1c, the resulting ionoacoustic signals are in the MHz range with pressure amplitude up to 100 Pa, depending on the proton pulse duration and charge^20^. However, clinical proton beams result in larger axial dimensions due to range straggling. Therefore, we used a ripple filter to spread-out the proton beam, in order to be closer to pre-clinical and clinical scenarios. The ripple filter is made of a sawtooth structure in aluminum alloy (represented in supplementary Fig. S1). As the accelerated protons penetrate different thickness of aluminum, exhibiting higher stopping power than water, they stop at different ranges in the water tank, resulting in an axial expansion of the Bragg peak volume. Furthermore, protons scatters more in aluminum than in water, leading to a subsequent increase of the proton beam lateral dimensions, as illustrated in Fig. 1d. As a consequence, the amplitude and frequency of the ionoacoustic pressure waves reduce, reaching the 100kHz to 200kHz frequency range, as depicted in Fig. 1e.

### Impulse response with mono-energetic and spread-out proton beam

#### Measurements without ripple filter

**Figure 2:**
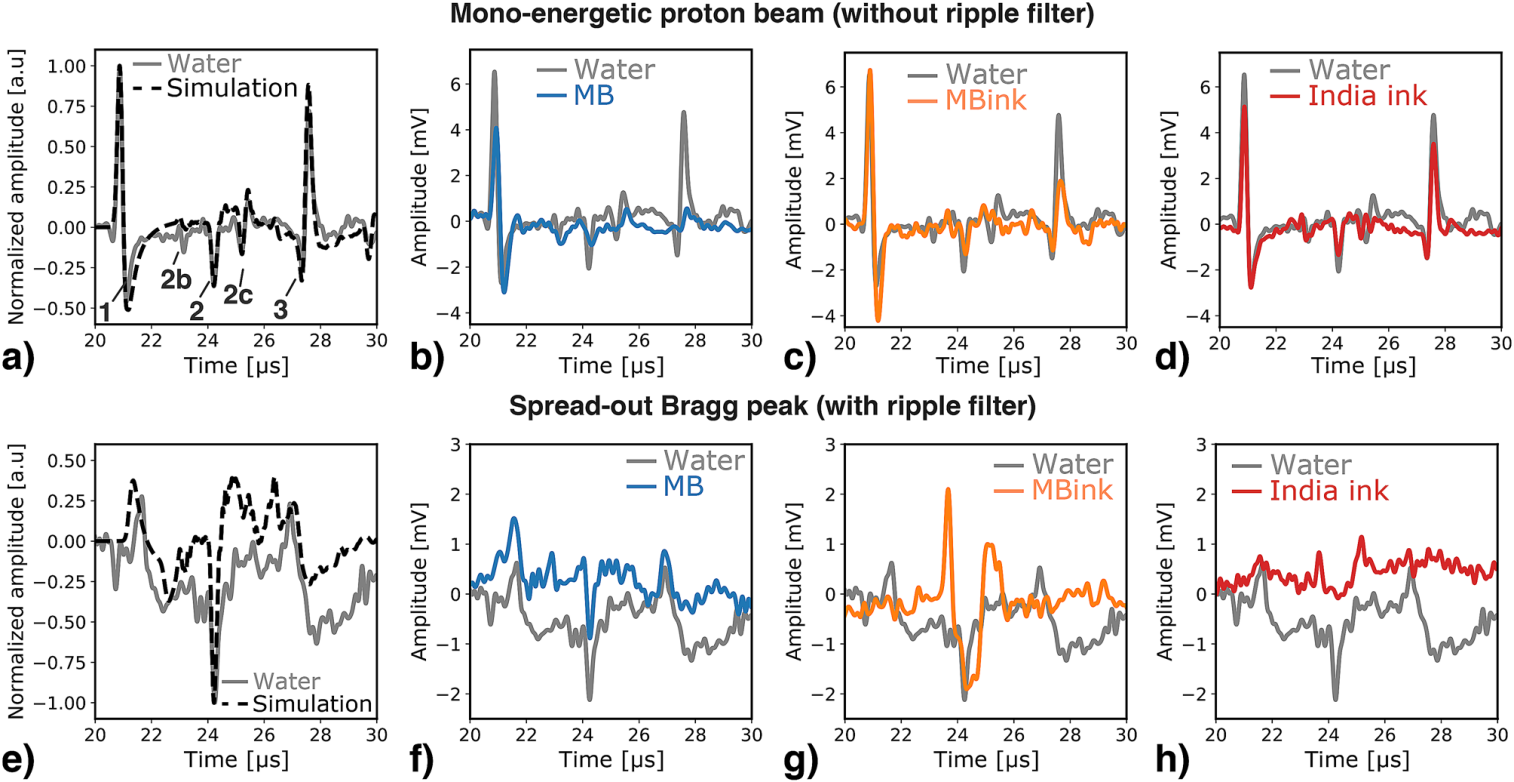
Ionoacoustic impulse response. Single-pulse $(200 \,\hbox {ns})$ measurements from mono-energetic 22 MeV proton beam, in water (gray) compared to (**a**) simulated signal (black dashed line), with (**b**) SonoVue microbubbles (blue), (**c**) SonoVue microbubbles in water/India ink mixture and (**d**) diluted India ink solution. The ionoacoustic signal is composed of a direct signal (1) arriving at $\hbox {t}={21}\,{\upmu \hbox {s}}$ (distance sensor to Bragg peak = 31 mm), pulse (2b) and its reflection (2c) from the CA phantom entrance and pulse (2) from the water tank entrance window at $\hbox {t}={24.3}\,{\upmu \hbox {s}}$ (distance sensor to entrance window $\simeq 36 \,\hbox {mm}$) and (3) the reflection of the direct signal (at $\hbox {t}={27.6}\,{\upmu \hbox {m}}$ corresponding to the 4.9 mm range of the proton beam). Ionoacoustic single-pulse (200 ns) measurements from the spread-out Bragg peak, in water (gray) compared to (**e**) simulated signal (black dashed line), with (**f**) microbubbles (blue), (**g**) microbubbles in water/India ink mixture and (**h**) diluted India ink solution. All the experimental measurements have been averaged over 3000 acquisitions and filtered using PyWavelets Python package.

Figure 2a–d shows the ionoacoustic signals obtained for a single proton pulse of 200 ns from the mono-energetic beam, i.e., without ripple filter. The signal is composed of three characteristic pulses, noted 1–3 in Fig. 2a. The first pulse (1) observed at ${21}\,{\upmu \hbox {s}}$ is the direct signal, resulting from the sharp dose gradient in the Bragg peak region whereas (3) is its reflection at the interface between the polyimide film window and air. The strong interface between air and the film induced discontinuity in the initial pressure distribution, resulting from energy deposition variation (more energy being deposited in the film) and a higher energy to pressure conversion (Grüneisen parameter) in polyimide than in air. As a consequence, an ionoacoustic wave is produced when the proton beam penetrated the water tank. In a similar manner, the CA phantom is closed on both sides by polyimide films. Therefore, it results in an additional discontinuity when the proton beam enters the phantom which is at the origin of the pulse (2b) and its reflection (2c).

Focusing on the microbubbles CA presented in Fig. 2b,c, the phase of the direct signal is shifted i.e., a change in the signal bipolarity with an increase of the negative rarefaction pulse is observed for both CA along with a decrease of the positive compression pulse for the microbubbles in water. Additionally, oscillations are observed at the end of the direct signal (at ${21.7}\,{\upmu \hbox {s}}$). It should be noted that the amplitude and frequency of the window signals (2) change with the presence of microbubbles and the direct signal reflection amplitude (3) reduces by 80%. The latter finding is attributed to acoustic impedance mismatch between the water and microbubble-filled phantom. The signal shape is preserved when only using India ink (Fig. 2d). Thus, phase shift and oscillation suggest a microbubble response to the ionoacoustic excitation. The former resulting from the superposition of the ionoacoustic pressure and the microbubble response induced pressure and the latter being a direct observation of the microbubble oscillations.

#### Measurements with ripple filter

Figure 2e–h depicts the ionoacoustic signals resulting from the energy deposition of the spread-out proton beam, i.e., with ripple filter. As already illustrated in Fig. 1e, the spread-out Bragg peak results in a lower signal amplitude and frequency. Since the pulse produced at the water tank entrance window (at $\hbox {t}={24.3}\,{\upmu \hbox {s}}$) is induced by interface discontinuity, it is almost not affected by the change in the dose distribution when spreading-out the Bragg peak volume. Consequently, its amplitude remains almost the same as with mono-energetic beam and is of higher amplitude than the direct signal from the Bragg peak, for the investigated setup. Note here, the simulated signal shown in Fig. 2e takes into account the electrical impulse response of the ultrasonic detector, leading to a distortion of the signal shape compared to the one presented in Fig. 1e. The low signal-to-noise ratio resulting from the drop of ionoacoustic pressure amplitude hampers the full interpretation of the measurements. However, it can be noticed that similar ionoacoustic signals (shape and amplitude) are obtained for the microbubble (Fig. 2f) and the measurement in water. The microbubbles dispersion in water/India ink mixture results in a noticeable increase of the signal amplitude, as illustrated in Fig. 2g. However, the enhancement is located at the entrance of the CA phantom and the information on the Bragg peak region (direct signal) seems lost. In a similar way, using the India ink alone results in a decrease of the direct signal amplitude and only the pulses from the CA phantom are visible, as showed in Fig. 2h.

### Response to 25-cycle burst using a mono-energetic proton beam

#### Evolution in time-domain

**Figure 3:**
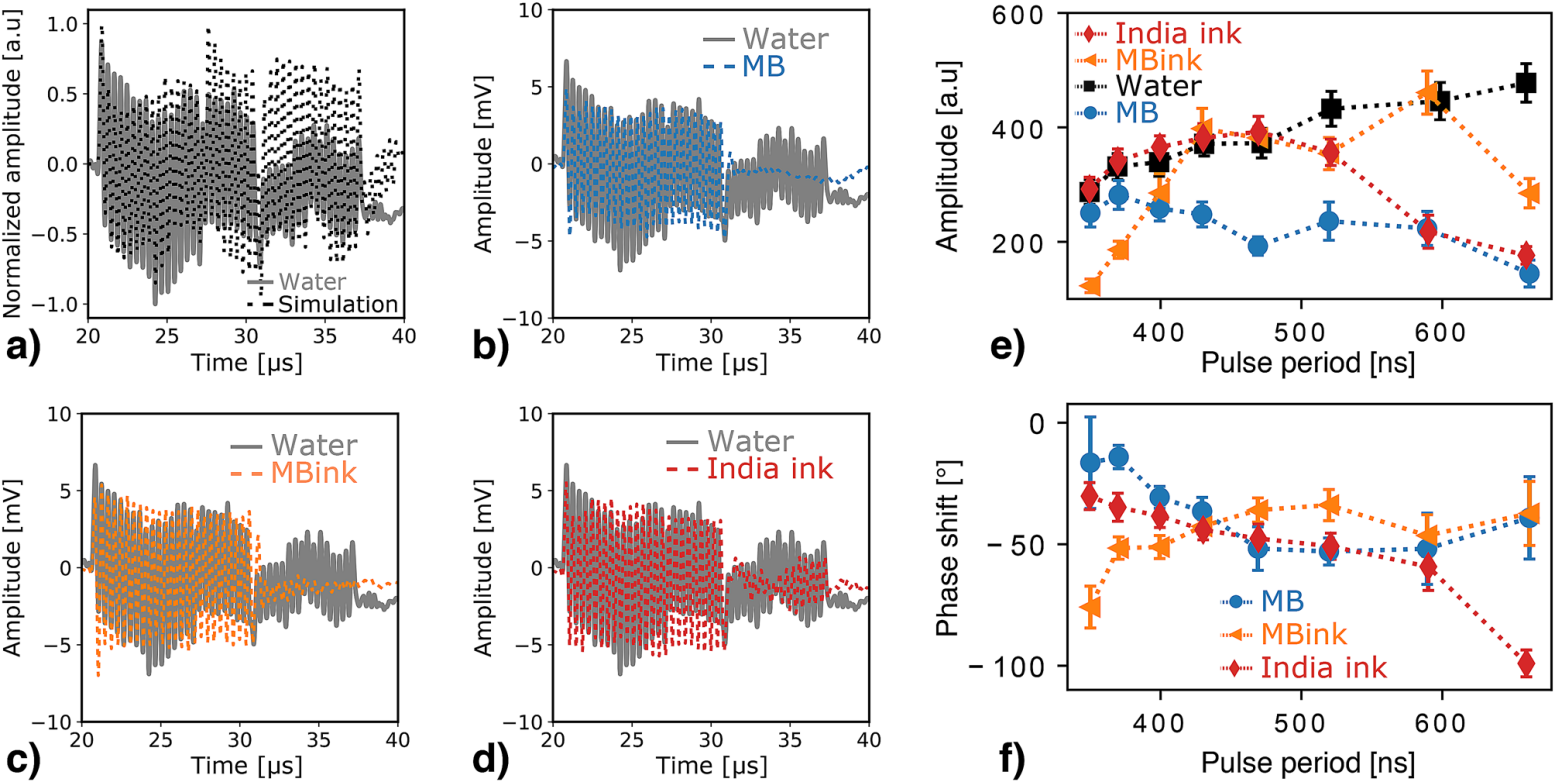
Evolution of the ionoacoustic signals with the pulsing period of the mono-energetic proton beam. Measured signals for a 25-cycle proton pulse of 400 ns period in water (gray) compared to (**a**) simulated signal in water (black dashed line), with (**b**) SonoVue microbubbles (blue), (**c**) SonoVue microbubbles / India ink mixture (orange), (**d**) India ink alone (red). All the experimental measurements have been averaged over 3000 acquisitions. (**e**) Evolution of the signal amplitude with the pulse period. The amplitude was extracted from the Fourier transform signals at the temporal excitation frequency to disentangle the high and low frequency components of the signal. (**f**) Phase shift as a function of the pulsing period between the signals measured in water and with CA. All the experimental measurements have been averaged over 3000 acquisitions and filtered using PyWavelets Python package. All error bars correspond to measurement uncertainties obtained from 30 consecutive measurements and given with a confidence level of 99%.

The microbubble resonance was investigated by increasing the number of cycles to 25 and the pulse frequency was varied on a range corresponding to the expected microbubble resonance frequency (period from 350 ns to 660 ns). Figure 3a–d show the results obtained for the mono-energetic proton beam and 400 ns period, i.e., 25 pulses of 200 ns separated by 200 ns time off, as illustrated in Fig. S3 of the supporting information. In this configuration, the temporal frequency imposed by the proton pulse time profile i.e., 25-cycle burst, is in the same frequency range (MHz) as the spatial frequency i.e., induced by the dimension of the Bragg peak volume, as illustrated in supplementary Fig. S4.

For the considered pulse period in water, the maximum/minimum amplitude successively decreases and increases as follow: it decreases from ${21}\,\hbox {to} \,{24.3}\,\upmu \hbox {s}$, increases from ${24.3}\,\hbox {to}\,{28}\,\upmu \hbox {s}$ and again decrease from ${28}\,\hbox {to}\,{31}\,\upmu \hbox {s}$, as depicted in Fig. 3a. The obtained pattern is the result of interferences between the 25 direct signals emitted for each single proton pulse composing the burst, the pressure waves produced at the different interfaces (CA phantom and water tank entrance) and subsequent reflections, as illustrated in Fig. S4. In detail, the first decrease can be explained by the sum of the individual direct signals. Indeed, the negative part of the first pulse generated overlaps with the positive part of the second pulse, resulting in an amplitude decrease. As a consequence, the evolution of the signal amplitude over the first cycles only depends on the frequency content of the ionoacoustic signal i.e., spatial frequency given by the dose, and the cycling period of the proton pulse i.e., temporal frequency and duration between two consecutive pulses. At ${24.3}\,\upmu \hbox {m}$, the window signal from the water tank starts to interfere with the direct signal, leading to an additional drop in amplitude, later compensated by interferences with bipolar CA entrance signals. The change in the signal shape at ${27.6}\,\upmu \hbox {s}$ corresponds with the arrival time of the reflection from the direct signal. Finally, the amplitude decrease and discontinuity observed at ${31}\,\upmu \hbox {s}$ is induced by the end of the 25-cycle excitation burst. Consequently, this additional low frequency pattern depicts the dose and relative Bragg peak position. Indeed, for a given proton burst time profile, the amplitude variation depends on the spatial frequency (dimension of the Bragg peak volume) and the low frequency pattern informs on the proton beam range (distance Bragg peak to water tank entrance window and time-of-flight between the direct signal and its reflection).

With CA, the signal amplitude remains almost constant over the excitation burst, as showed in Fig. 3b–d. The reduced amplitude of the reflections entailed by the acoustic impedance mismatch between water and CA may explain the constant amplitude after ${24.3}\,\upmu \hbox {s}$ and the clear signal end after the ${10}\,\upmu \hbox {s}$ excitation burst, while a longer tail is observed in water. This is particularly pronounced with the microbubbles (Fig. 3b), for which the signal amplitude drops to zero at ${31}\,\upmu \hbox {s}$ whereas low amplitude oscillations remain visible for microbubble combined with India ink and diluted India ink, as illustrated in Fig. 3c,d, respectively.

#### Evolution of the signal amplitude with the pulsing period

Figure 3e shows the evolution of the signal amplitude depending on the proton burst period and extracted in the frequency-domain at the excitation frequency (given by the proton pulse time profile). As it could be expected for the considered proton pulse periods, the ionoacoustic signal amplitude in water increases with the pulse duration until the temporal excitation frequency matches with the spatial frequency^20^. Therefore, the maximum of amplitude is obtained for proton pulse duration around the so-called stress-confinement limit i.e., the time it takes for the acoustic wave to propagate through the heated volume, here around ${300}\,\upmu \hbox {s}$ considering the ${430}\,\upmu \hbox {m}$ FWHM axial width of the 22 MeV proton beam in water. In general, the signal amplitudes measured with the CA are lower or equivalent to the ones obtained in water. This may be partially attributed to the acoustic impedance mismatch between water and CA, as confirmed from ultrasound pulse-echo measurements and found to be particularly enhanced in case of microbubbles (see Fig. S5 of the supporting information). Using the microbubbles alone, the amplitude is always lower than in water and remains almost constant independently of the pulse period, with a noticeable drop at ${470}\,\upmu \hbox {s}$. In general, the signal amplitude increases with the pulse period when adding India ink to the microbubbles, with amplitudes similar to the ones in water for pulse periods longer than ${430}\,\upmu \hbox {s}$ and amplitude drops at ${520}\,\upmu \hbox {s}$ and ${660}\,\upmu \hbox {s}$. For the India ink, the amplitude follows the evolution of the one in water for a pulse period up to ${470}\,\upmu \hbox {s}$ and unexpectedly decreases for longer proton pulse duration.

#### CA induced phase shift

Figure 3f depicts the phase difference between the measurements in water and those with CA at the excitation frequency. Note that, as previously mentioned, for the microbubble-based CA the phase shift refers to a change in the shape of the ionoacoustic signal, whereas for ink alone the phase shift is likely due to the different speed-of-sound in the two media (water and India ink diluted in water) and consequent variation of the ionoacoustic signal wavelengths and time-of-flight difference. For the microbubbles, the phase shift decreases from almost ${0}^{\circ }$ to ${-50}^{\circ }$ for pulse periods ranging from ${350}\,\hbox {to}\,{470}\,\upmu \hbox {s}$, respectively, and remains constant for longer pulses. It is interesting noting that the period from which the phase shift reached the plateau region also corresponds with a drop of the signal amplitude, suggesting the amplitude with the microbubbles is dictated by interferences between the ionoacoustic signal and the microbubble contribution. The phase shift evolution is reversed when India ink is added to the microbubbles but behaves similarly with a plateau region also observed from ${470}\,\upmu \hbox {s}$. Finally, for the diluted India ink, the phase shift decreases almost linearly.

### Response to 25-cycle burst using a spread-out proton beam

#### Evolution in time-domain

**Figure 4:**
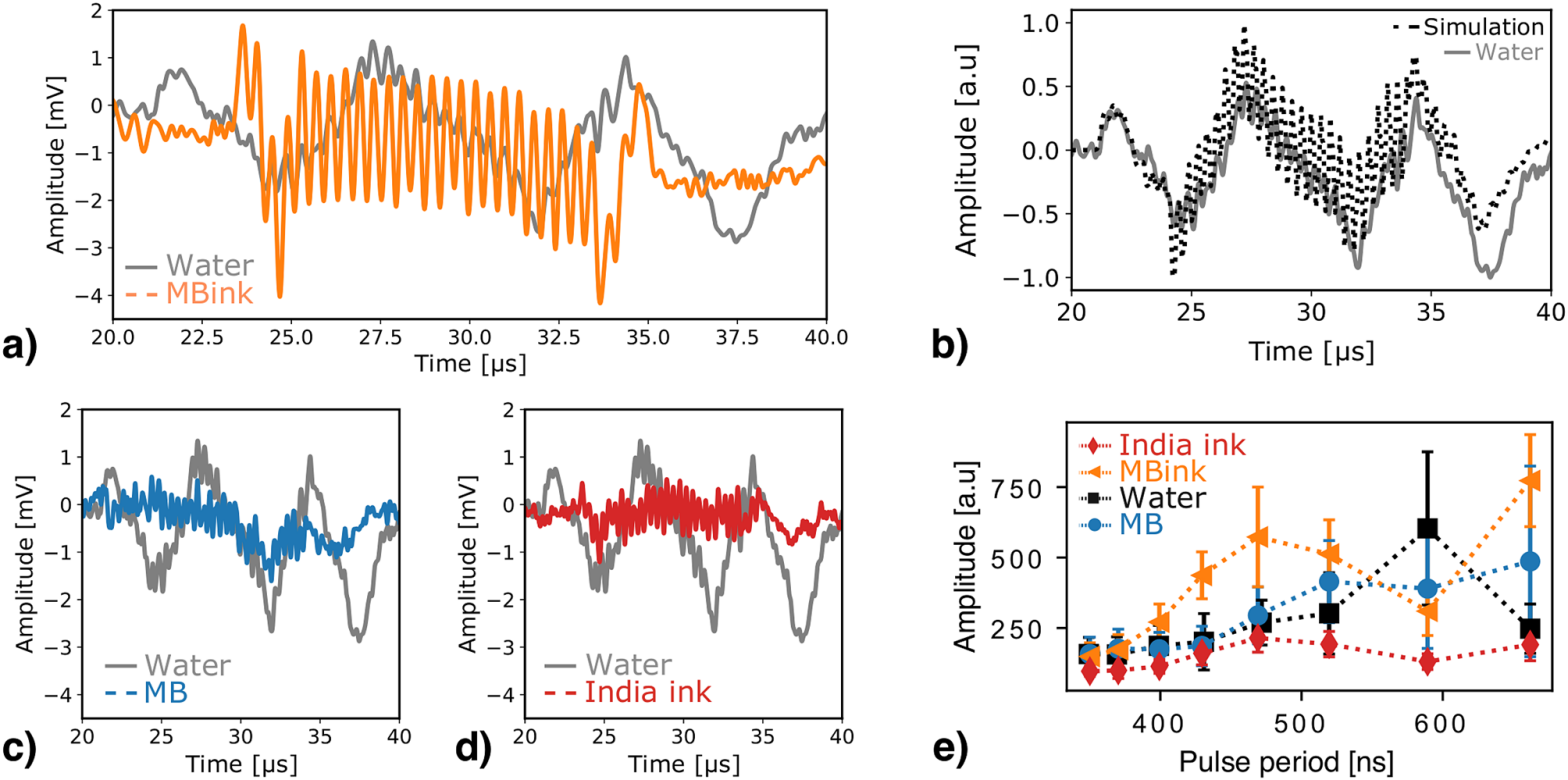
Evolution of the ionoacoustic signals with the pulsing period of the spread-out Bragg peak. Ionoacoustic signals measured for the spread-out proton beam and 25-cycle proton pulse of 400 ns period in water (gray) compared to (**a**) SonoVue microbubbles / India ink mixture in water (orange), (**b**) simulated signal in water (black dashed line), (**c**) SonoVue microbubbles in water (blue), (**d**) India ink in water (red). All the experimental measurements have been averaged over 3000 acquisitions. (**e**) Evolution of the signal amplitude for irradiation with the ripple filter depending on the pulse period. The amplitude was extracted from the Fourier transformed signals at the temporal excitation frequency to disentangle the high and low frequency components of the signal. All the experimental measurements have been averaged over 3000 acquisitions and filtered using PyWavelets Python package. All error bars correspond to measurement uncertainties obtained from 30 consecutive measurements and given with a confidence level of 99%.

Similar to the mono-energitic case, here also the microbubble resonance was investigated by increasing the number of cycles to 25 and varying the proton pulse frequency. Figure 4a–d shows the results obtained for the spread-out proton beam and a 400 ns period. In these conditions, the temporal frequency imposed by the proton pulse time profile is in the MHz frequency range, whereas the spatial frequency is of the order of a few hundreds of kHz frequency. Like the measurements in water with the mono-energetic beam, the signal amplitude varies with a low frequency pattern dictated by the interferences between the different ionoacoustic signals, reaching a first minimum around ${24.3}\,\upmu \hbox {s}$ followed by a maximum at ${27.6}\,\upmu \hbox {s}$ , as illustrated in Fig. 4b. The direct signal (from ${21}\,\hbox {to}\,{24.3}\,\upmu \hbox {s}$) does not capture the MHz oscillations induced by the proton pulse time profile due to the mismatch of the spatial and temporal frequency contents. Furthermore, since the entrance window signals preserve their shape and MHz frequency content even with the ripple filter (see Fig. 1e), high frequency oscillations are observed on the ionoacoustic measurements in water from ${24.3}\,\upmu \hbox {s}$. As previously observed, the microbubbles with India ink lead to a noticeable enhancement of the window signal amplitude, but the information on the direct signal is lost, as can be seen in Fig. 4a. Furthermore, the phase of the CA phantom window signal is shifted compared to the water tank entrance window signal, the former being a positive pulse and the latter a negative one (see Fig. 2g). As a consequence, the first two pulses observed for the microbubbles with India ink are positive followed by bipolar oscillations, resulting from interferences between the two window signals. Similar trends are observed for the microbubbles in water and diluted India ink. In both cases, the high frequency comes from the entrance window almost without any low frequency pattern, as showed in Fig. 4c,d, respectively.

#### Evolution of the signal amplitude with the pulsing period

The evolution of the signal amplitude with the proton pulse period is showed in Fig. 4e. In water, the amplitude increases as a function of the pulse duration with a peak amplitude for 590 ns pulse period likely corresponding to the period for which the temporal excitation frequency matches with the water tank window frequency. Both measurements with the microbubbles and India ink alone do not significantly enhance the signal amplitude which increases similarly to the one in water for pulse periods shorter than 470 ns. For longer pulses, the amplitude with microbubbles remains almost constant without any peak at 590 ns, suggesting the signal is not affected by the water tank entrance window signal as for water. With the India ink, the signal amplitude reaches its maximum value for 470 ns pulse period and decreases for longer pulses, as previously observed for the measurements without ripple filter. Finally, the microbubbles with India ink demonstrate an enhancement of up to 200% of the signal amplitude compared to water, with a drop of amplitude for a pulse period around 590 ns, corresponding with maximum amplitude of the signal in water. This finding could be understood as the result of destructive interferences between the water tank entrance window signal and the one from the CA phantom window. When the water tank entrance window signal reaches an amplitude similar to the one of the CA phantom window signal, whereby the two window signals cancel out (see Fig. S6 of the supporting information) explaining the amplitude drop observed in Fig. 4e. We assume this to be a first evidence of an additional photoacoustic effect that may contribute to the ionoacoustic pressure waves.

## Discussion

### Experimental campaign at pre-clinical proton beam energy

CA have the potential to increase the amplitude and frequency of ionoacoustic signals. This could be a breakthrough in the development of ionoacoustic range verification for pre-clinical and clinical proton beam diagnostic currently hindered by the detection limits. Our different measurements reveal evidences of microbubble-based CA excitation with, among others, additional oscillations, phase shift and different evolution of the amplitude with the proton pulse period compared to measurements in water alone. However, our results do not show an enhancement of the signal amplitude due the presence of microbubbles themselves. When the temporal and spatial excitation frequency of the ionoacoustic signal are in agreement, i.e., measurements without ripple filter, the amplitude is reduced in the presence of microbubbles. This could be explained considering the acoustic impedance mismatch between the microbubble CA and water which results in an amplitude decrease of the pressure received by the ultrasonic sensor compared to the pressure amplitude in the vicinity of the Bragg peak. In other words and especially for the shortest pulse duration for which similar amplitudes are measured in water and with the microbubbles, the pressure might actually be enhanced by the microbubbles, but we cannot measure it due to reduced acoustic transmission between the phantom and the surrounding water.

Moreover, we showed that combining microbubbles and India ink results in an amplitude increase of up to 200% of the signals obtained from spread-out dose distribution i.e., measurements with ripple filter, originating from the entrance of the CA phantom. For the mono-energetic proton beam, the signal amplitudes are lower for the microbubbles and India ink than the microbubbles alone for a proton pulse period up to 430 ns and increase for longer pulses. This noticeable discrepancy in the microbubble behavior might either be explained by an intrinsic change in the microbubble oscillation response due to the additional ink in the surrounding medium or additional contributions such as photoacoustic effect. Photoacoustics could be induced by the energy deposition, resulting from the absorption of optical photons produced in water along the proton beam axis.

Water luminescence has already been reported for proton beams at energy lower than the Cherenkov light threshold ($<{480}{\,\hbox {MeV}}$ for proton beam in water), showing good agreement between the measured optical photon distribution and proton dose deposition. To a minor extent, Cherenkov light is emitted from secondary products, resulting from ionization and nuclear interaction of the primary proton beam and the irradiated water. Notably, secondary electrons from ionization losses of the protons, in addition to Compton scatter electrons and electron/positron pair production from prompt gamma photons can exceed the Cherenkov light threshold (around 260 keV for electrons in water), depending on the incident proton beam energy. However, Cherenkov light from secondary electrons leads to a spread-out optical photon distribution and maximum of the light fluence upstream to the Bragg peak. These characteristics are not in agreement with the experimental observations, highlighting additional luminescence process. To the best of our knowledge, there is no clear consensus on the origin of the water luminescence which might be attributed to radicals produced in water during the irradiation or resonance of the electromagnetic dipole of water molecules^21^.

### Simulation of the water luminescence and resulting photoacoustic pressure

**Figure 5:**
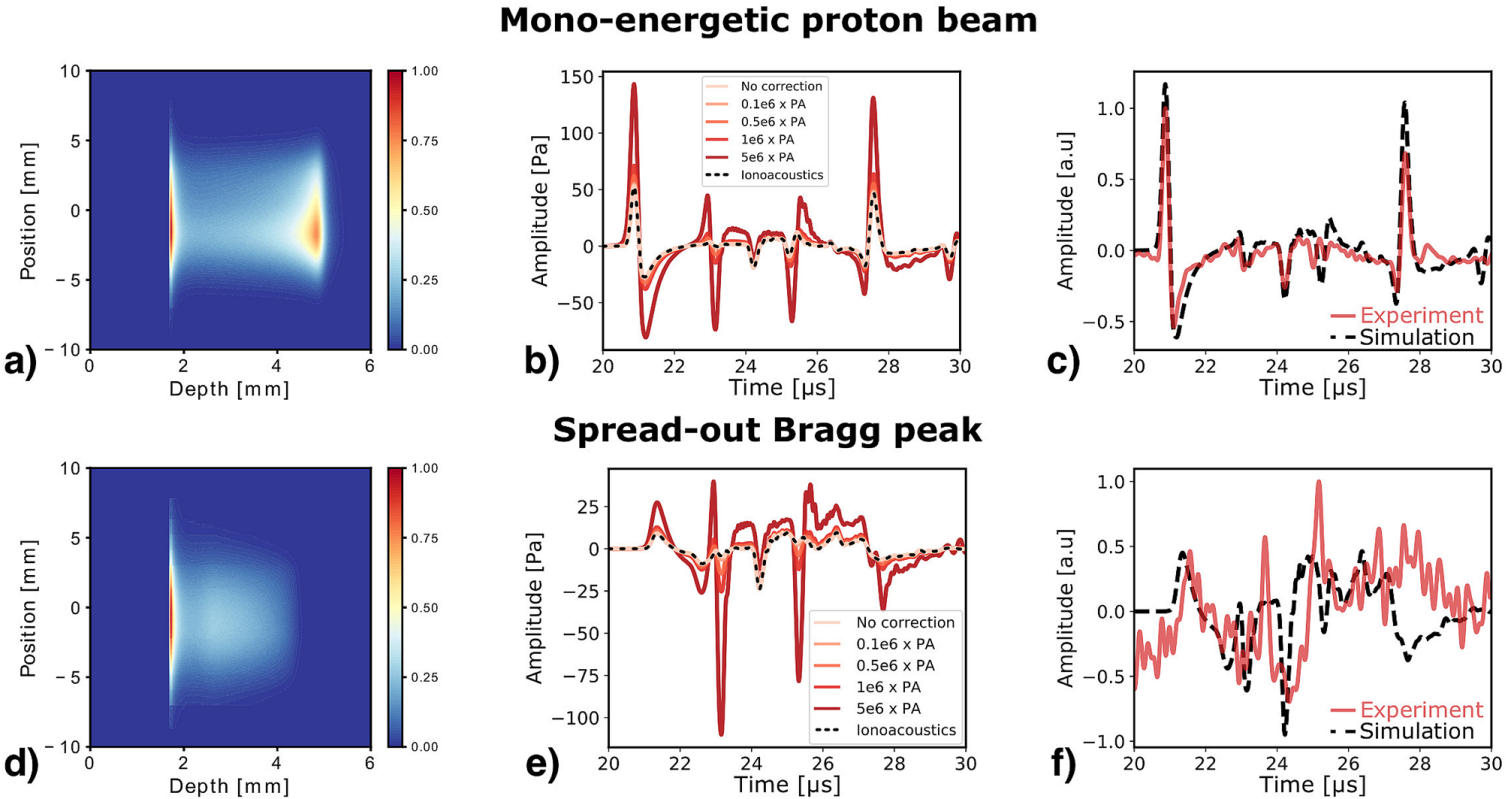
Water luminescence simulations and resulting pressure. (**a**) Energy deposition of the optical photons for the mono-energetic proton beam obtained from Fluka2021.1beta and (**b**) resulting ionoacoustic signal depending on the correction factor applied to the photoacoustic contribution (PA). (**c**) Comparison between the measurement with India ink using the mono-energetic beam and the simulation with a correction factor of ${5\times 10^{5}}$. (**d**) Energy deposition of the optical photons for the spread-out Bragg peak obtained from Fluka2021.1beta and (**e**) resulting ionoacoustic signal depending on the correction factor applied to the photoacoustic contribution. (**f**) Comparison between the measurement with India ink using the spread-out Bragg peak and the simulation with a optical photon correction factor of ${5\times 10^{5}}$. All the experimental measurements have been averaged over 3000 acquisitions and filtered using PyWavelets Python package.

A simulation study was performed in order to assess if the postulated photoacoustic effect is the origin of our experimental observations. Figure 5 shows the results obtained by reproducing water luminescence from scintillation light, as suggested by Yabe et al.^19^. It should be noted that, the simulations performed do not model the Physics responsible for the water luminescence (which remains to be clarified), but is only using the equivalent model proposed by Yabe et al., which has been shown to give good agreement between simulations and experiments for a given setup and imaging modality. Figure 5a,d depict the expected energy deposition resulting from the absorption of the optical photons for the mono-energetic proton beam and the spread-out Bragg peak, respectively. In both cases, a sharp gradient is observed at the interface between water and the CA phantom (at 1.6 mm), resulting from the high absorption in India ink compared to water (less than ${1}{\,\hbox {mm}^{-1}}$ in water and ${3.7\times 10^{3}}{\,\hbox {mm}^{-1}}$ for 1% of India ink at 632.8 nm^22^). From 0–1.6 mm, the energy deposited in water by the optical photons is negligible compared to the energy deposited above 1.6 mm (in India ink) which follows the proton dose distribution showed in Fig. 1b. Such a distribution seems in agreement with our experimental observations, as the prominent sharp gradient at the interface would lead to strong high frequency acoustic emission.

Figure 5b,e shows the pressure resulting from the superposition of the ionoacoustic (from the proton beam energy deposition) and photoacoustic (from the optical photon absorption) effects. Applying the suggested 0.1 photon/MeV light yield results in a very low pressure amplitude (inferior to 0.1 mPa) from the photoacoustic effect which is not sufficient to interfere with the ionoacoustic signals (which is of the order of a few hundreds of Pa). To have a notable impact on the ionoacoustic pressure wave, i.e., amplitude increase and signal distortion, the photoacoustic pressure would need to be 5–6 orders of magnitude higher, as illustrated in Fig. 5b,e, equivalent to a light yield of around ${5\times 10^{4}}$ photons/MeV. In these conditions, the photoacoustic contribution induces an increase of the signal amplitude, sufficient to modify the interference pattern observed when using 25-cycle burst. The simulated pressures are compared to the experimental measurements in Fig. 5c,f, with a photoacoustic signal increased by a factor ${5\times 10^{5}}$ compared to its original literature value considering a light yield of 0.1 photon/MeV. The correction factor was chosen in order to match the amplitude of the signal produced in this work at the CA phantom entrance relative to the direct signal and water tank entrance window. Signals obtained with intermediate correction factors are shown in supplementary Fig. S10. Including the photoacoustic contribution allows for a better match between the simulated pressure and measurement for the mono-energetic beam, increasing the relative amplitude of the CA entrance window signal. However, the simulation suggests an increase of the ionoacoustics direct signal amplitude compared to the measurement in water which was not observed experimentally. The simulation for the spread-out Bragg peak including a photoacoustic contribution tends to explain the experimental observations, with an enhanced pulse arriving from the CA entrance window, without noticeable increase of the direct signal amplitude. However, the shape and polarity of the CA entrance signal is not fully reproduced. Note that the difference on the arrival time of the direct signal between the simulation and the experiment is also observed in water (cf. Fig. 2e) and is attributed to an inaccuracy of the proton beam model in the Monte Carlo simulation due to proton beam instability during the experiments.

The discrepancies in the shape of the signals between the simulations and experiments might be explained by an inaccurate model of the CA properties. Indeed, for the simulations we assumed the CA to have the same characteristics as water. In reality, the additional India ink likely alters the medium properties (density, speed-of-sound and Grünensein parameter) sufficiently to notice the changes compared to the measurement in pure water. The acoustic impedance mismatch between CA and water was also not modeled in the simulations. This can partially explain the observed discrepancy in the direct signal amplitude, i.e., the increase of the direct signal amplitude which is not observed from the experiments. However, from the ultrasound pulse-echo measurements and evolution of the ionoacoustic signal time-of-flight, the acoustic property changes do not seem to be large enough to entirely explain the observed difference in the signal shape and do not justify the 5–6 orders of magnitude difference on the postulated light yield compared to the value previously reported by Yabe et al.

For a given proton pulse time profile, the shape of the ionoacoustic (and potential photoacoustic) signal mostly depends on the spatial gradients of the absorbed energy, whereas the amplitude varies to a lesser extent with the gradients and increases linearly with the energy deposition. Therefore, the disagreement between the simulations and experiments may suggest that the approximate water luminescence using a scintillation model as previously suggested does not accurately reproduce the overall optical photon distribution, which could actually be more localized in space than what is obtained from scintillation simulations. Note, the simulation studies previously reported have been focused on modelling the portion of the optical photons propagating on an axis perpendicular to the proton beam axis and detected on a plane parallel to the beam axis, but did not investigate the optical photon three-dimensional distribution. Hence, assuming a certain directivity for the optical photons from water luminescence, which could be different to the isotropic propagation of the photons modelled using scintillation, the light yield would need to be changed to reproduce the same optical photon fluence at the detector surface. For instance, if the photons from water luminescence mostly propagated forward as for the Cherenkov light, the light yield would need to be increased in order to obtain the same amount of photons propagating on an axis perpendicular to the proton beam axis. Such a directivity could then explain the discrepancies we observe. Furthermore, we assumed the optical photon production to be the same in India ink as in water, but it is more realistic to consider the light yield in ink to be higher than in water^18^. Therefore, the increase of optical photon production in India ink compared to water would lead to an increase of the energy absorption and distortion of the sharp gradient at the CA phantom entrance. A similar effect would be also expected for the polyimide foil at the entrance of the CA phantom which is known to produce scintillation light with a sufficiently high light yield to explain the 5–6 orders of magnitude difference observed. Hence, our experimental observations may result from a mixed contribution of proton-induced luminescence and scintillation light from the polyimide film.

### Use of contrast agents with clinical proton beams

The investigated CA mainly enhance the acoustic emission at the interface between the targeted volume (where the CA is concentrated) and the surrounding medium. However, accurate proton dose monitoring requires precise measurement of the direct signal from the Bragg peak, in order to deduce the distance from the sensor to the dose maximum and the spatial distribution of the proton dose deposition. Therefore, the clinical benefits of CA and new opportunities they may bring for ionoacoustics-based range verification remain to be investigated. Assuming light yields similar to those deduced from our experiments, the enhanced ionoacoustic signals were investigated for clinical proton beams delivered from two different particle accelerator technologies. First, for synchro-cyclotrons which result in a pulsed proton beam, with Gaussian proton pulses in the order of a few ${\,\upmu \hbox {s}}$, particularly suitable for ionoacoustic measurements^8^. Second, for synchrotron accelerators providing continuous proton beams that are consequently, in first approximation, not suitable for ionoacoustics. However, they still have a temporal micro-structure in the 5–10 MHz range, which could be enhanced thanks to the CA.

**Figure 6:**
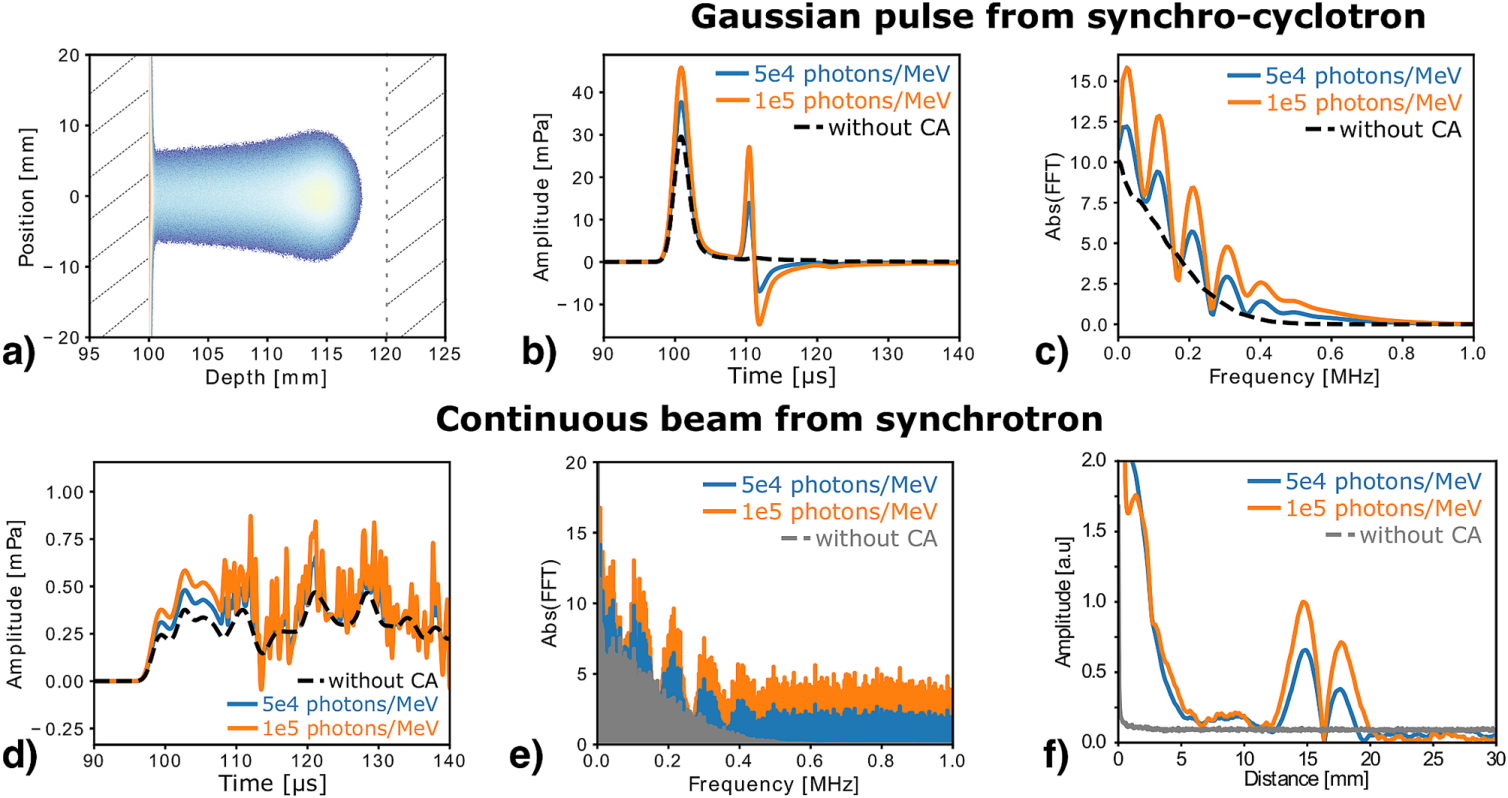
Simulated photo-enhanced ionoacoustic signal from clinical proton beams in tumor and liver tissue. (**a**) Energy deposition of the optical photons obtained from Fluka2021.1beta, the hatch area represents the liver. Ionoacoustic signals obtained from synchro-cyclotron (${2.5}{\,\upmu \hbox {s}}$ Gaussian pulse) without CA (black) or with CA and photoacoustic pressure corresponding to a light yield of ${5\times 10^{4}}$ photons/MeV (blue) and ${1\times 10^{5}}$ photons/MeV in (**b**) time and (**c**) frequency-domain. Ionoacoustic signals obtained from synchrotron (50 ms burst with a MHz micro-structure) without CA (black) or with CA and photoacoustic pressure corresponding to a light yield of ${5\times 10^{4}}$ photons/MeV (blue) and ${1\times 10^{5}}$ photons/MeV in (**d**) time and (**e**) frequency-domain. (**f**) Fourier transform of the filtered spectra show in (**e**), depicting the distance from the Bragg peak to the entrance of the tumor, assuming a speed-of-sound of ${1556.9}{\,\hbox {ms}^{-1}}$.

Figure 6a shows the energy deposition of the optical photons produced by a 130 MeV proton beam, with the Bragg peak located inside a 2 cm target (at 11.4 cm along the beam axis, 14.49 mm after entering the target). The phantom geometry was chosen to be similar to the previous experiments, whereas the medium properties were set to correspond with an hepatic tumor lesion and surrounding liver tissue. Figure 6b shows the ionoacoustic signals obtained with and without CA. The corresponding spectra are presented in Fig. 6c. The CA leads to an increase of the direct signal amplitude (at $\hbox {t}={100}{\,\upmu \hbox {s}}$) and induces a second pulse (at $\hbox {t}={110} {\,\upmu \hbox {s}}$) at the interface between the tumor and liver where the beam enters the targeted volume. Consequently, in frequency-domain, the spectrum of the signal with CA is pushed to higher frequency and oscillates with a pulsing period (beats) related to the distance between the Bragg peak and tumor entrance.

The ionoacoustic signals obtained considering the temporal micro-structure of synchrotrons are shown in Fig. 6d,e in time and frequency-domain, respectively. Using the CA allows to capture the temporal micro-structure of the proton beam which is not observed on the signal without CA. Similar to the signal obtained from synchro-cyclotron, the spectra depict oscillations of frequency till 500 kHz followed by a broad frequency response for higher frequencies. Note also the very low amplitude of the ionoacoustic signals, which remains below 1 mPa. Although current progress in the development of high-sensitivity ultrasonic transducers should allow detection of such a low amplitude^23^, the ambiguous signal shape and limited possibility to average may hamper the time-of-flight analysis generally used to retrieve the Bragg peak position relative to the sensor. Alternatively, long continuous bursts are favorable to accurately analyze the data in the frequency-domain, offering both high frequency resolution and high signal-to-noise ratio (the signal being averaged over time).

Therefore, the CA which induces spectrum beats due to the additional pressure generated at the target entrance seems beneficial to ionoacoustic range verification using a continuous beam. Indeed, this information can be used to retrieve the distance between the Bragg peak and target entrance. Figure 6f shows the Fourier transform of the spectrum beat transposed to the spatial domain, assuming a constant speed-of-sound in the tumor. As can be seen in Fig. 6f, the beat in the ionoacoustic signal spectrum corresponds to a distance ranging from 12.5 to 20 mm which is in good agreement with the distance from the Bragg peak to the tumor edge. Furthermore, the maximum of amplitude of the signal beats correspond to a distance of 14.88 mm and 14.68 mm for a light yield of ${5\times 10^{4}}$ photons/MeV and ${1\times 10^{5}}$ photons/MeV, respectively. This corresponds to a sub-millimeter accuracy on the distance from the Bragg peak to the entrance of the tumor with an error relative to the proton beam range lower than 0.3% (0.27% and 0.13% , respectively). The promising results are also confirmed when moving the tumor along the beam axis (see supplementary Fig. S11) for which the relative error remains below 0.3% for all the investigated phantom geometries.

### Conclusion and future contrast agent development

In conclusion, we experimentally showed that combining India ink to microbubbles increases the amplitude of the ionoacoustic signals produced when a proton beam enters the targeted volume (CA phantom). The effect may reveal the absorption of optical photons along the proton beam axis. It is assumed that, in water, the optical photons generated are spread-out because of the low absorption in the medium. With optical absorbers, such as India ink, the energy resulting from their absorption is more localized, giving rise to acoustic pressure in the same order of magnitude as the one resulting from the energy deposition of the primary proton beam, the latter being enhanced in the presence of microbubbles.

Further studies are required to elucidate the origin of the optical photons, which may arise from a mixed contribution of water luminescence and scintillation light, specific to the experimental setup used. Assuming sufficiently high optical photon fluence to produce relevant photoacoustic pressures consistent to our experimental observations, we showed that photo-enhanced ionoacoustics is beneficial for accurate range verification in proton beam therapy, resulting in a higher signal-to-noise ratio (due to the pressure enhancement) and intrinsic co-registration with the underlying anatomy (tumor edge visible on the ionoacoustic signal). More interestingly, this finding can also extend the use of ionoacoustics to a broader range of clinical accelerators. Currently, ionoacoustics is restricted to ${\,\upmu \hbox {s}}$ pulsed proton beams delivered from standard cyclotrons integrating custom-built pulsing system^10^ or synchro-cyclotrons^8^. To the best of our knowledge, our results show for the first time that accurate ionoacoustic range verification can be achieved at synchrotron facilities, using photo-enhanced ionoacoustics and frequency-domain data analysis.

Therefore, optimal ionoacoustic CA/signal enhancers could be based on functionalized microbubbles. In this case, the microbubbles will promote high frequency signals and the functional bubble shell could be optimized to enhance the photoacoustic effect and consequently the ionoacoustic signal amplitude. This can be done by incorporating both absorbing materials (enhancement of the optical photon energy deposition) and scintillation materials (enhancement of the number of photons produced). Furthermore, there is a growing interest for gold nanoparticles in photoacoustic imaging^24^ due to plasmonic resonance, contributing to photoacoustic signal enhancement. In this regard, gold nanoparticle-templeted microbubbles were investigated and shown to further enhance the signal dynamics^25^. Moreover, gold nanoparticles used as radiosensitizers also raised an interest in radiotherapy as they can enhance the treatment biological effectiveness^26^. Therefore, such agents already foreseen to be used in clinical routine could be combined for local treatment efficiency enhancement and accurate treatment monitoring, both beneficial for improving the clinical radiotherapy outcomes.

## Methods

### Proton beam characteristics

The 22 MeV proton beam was delivered by the Tandem accelerator of the Maier-Leibnitz-Laboratory in Munich (Germany). The proton beam was pulsed by means of a chopper system, resulting in square pulses with rise/fall times below 3 ns and pulse duration ranging from 10 ns up to a few ${\upmu \hbox {s}}$. During the experiments the chopper system was controlled by a function generator (33220A, Agilent Technologies Inc., USA) in order to vary the pulse period and number of cycles. Single proton pulse measurements were carried out with 200 ns pulses at a repetition rate of 10 kHz. The period of 25 cycle bursts was varied from 350 to 660 ns, with a duty cycle of 0.5 and a burst repetition rate of 10 kHz. The detailed proton burst structure is illustrated in Fig. S3 of the supplementary information. The function generator parameters were adjusted according to the synchronization output of the accelerator chopper system to ensure correct proton pulse time profiles. For each measurement, the trigger signal delivered by the chopper system was recorded and used to trigger the data acquisition system. The proton time profiles were measured prior to the experiments with the CA. The signal current generated by individual proton pulses inside a thin and fast silicon detector^27^ was recorded using a 4 GHz oscilloscope (LeCroy WaveRunner 640Zi, Teledyne LeCroy, USA) and time profiles were determined by deconvolution of the detector response function^28^. The pulse width at the function generator was varied from 140 ns to 300 ns and the deconvolved time profiles were compared to the signals obtained from the accelerator chopper system, confirming the reliability of the proton pulse duration obtained from the chopper system (see Fig. S7 of the supplementary information). The proton beam focus size was adjusted to be about ${4}{\,mm^{2}}$ and the dimensions were controlled by EBT3 films placed before the water tank (see Fig. S2). For the experiments, the number of particles per pulse was set between ${2.8 \times 10^{6}}$ and $5.6\times 10^{6}$ particles per pulse for pulse duration of 350 ns to 660 ns, respectively. The number of particles was deduced from the proton beam current, measured by a Faraday cup at the beam vacuum exit. For each setup and given proton pulse time structure, the ionoacoustic signals were measured over 3000 acquisitions. All the acquisitions were recorded individually and averaged offline during data analysis.

### Ionoacoustic measurements

For the measurements we used a 3.5 mm-diameter single-element reverse-fabricated CMUT probe. The transducer was designed to operate in emission-reception around 12 MHz^29^. The probe integrates a low-noise amplifier in order to optimize the performance in reception mode. The in-probe low-noise amplifier consists of a high input impedance, high-voltage protected, low-noise voltage buffer (MAX4200, Maxim Integrated, Sunnyvale, CA). The integrated front-end electronic was designed to operate in the 40 kHz to 15 MHz range in reception. We previously showed that the broadband capability of this detector allows to effectively measure ionoacoustic signals in the investigated frequency range without signal distortion induced by the sensor frequency response^30^. This unique feature motivated the choice of the CMUT probe for the experiments as it ensures a proper detection of both the kHz and MHz signals, as required by the measurements with ripple filter, whereas conventional piezo-composite-based transducers, typically characterized by narrower bandwidths, would have only captured one range of frequencies. Note that ionoacoustic measurements could have been performed with hydrophones to operate on such a large bandwidth but the CMUT probe also allows for ultrasound measurements in emission/reception to image the target. The signal received by the CMUT detector was amplified by 60 dB using a low-noise voltage amplifier (HVA-10M-60-B, FEMTO Messtechnik GmbH, Germany) and acquired through a digital oscilloscope (6404D PicoScope, Pico Technology Ltd., GB) at a sampling frequency of 156.25 MHz. In reception only, the CMUT was biased at 75% of its collapse voltage $(V_{collapse}={310}{\,\hbox {V}})$. For ultrasound measurements, both in emission and reception the bias voltage was maintained at 75% of the collapse voltage and a 70 ns-negative pulse of 5 V amplitude, delivered by a high frequency ultrasonic pulser (DPR300 JSR Ultrasonics, Imaginant Inc., USA) was sent to the transmission input of the CMUT probe.

### Contrast agent preparation

The contrast agent solutions were prepared right before irradiation. The microbubbles were obtained from a SonoVue preparation (SonoVue, Bracco Imaging S.p.A, Italy) vigorously shaken for one minute and later diluted in deionized water (${5.0\pm 0.1}$ ml of SonoVue microbubbles diluted in ${200\pm 1}$ ml of water, ${2.4\pm 0.1}$% microbubble concentration). The India ink based contrast agents were obtained from black drawing ink (Pelikan Fount India ink, Pelikan Group GmbH, Germany). For the microbubbles with India ink preparation, ${1.0\pm 0.1}$ ml of India ink was added into ${100\pm 1}$ ml of the microbubbles / water solution to obtain final concentrations of ${2.4\pm 0.1}$% of microbubbles and ${1.0\pm 0.2}$% of India ink. The India ink mixture was obtained from ${100\pm 1}$ ml of deionized water and ${1.0\pm 0.1}$ ml of India ink (${1.0\pm 0.1}$% ink concentration).

### Data analysis

For the time signals presented in Figs. 2, 3 and 4, datasets regrouping 3000 acquisitions were averaged and filtered using Wavelet decomposition from the PyWavelets Python package^31^. The filter parameters and threshold were manually adapted in order to preserve the signal shape and amplitude. We used a Daubechies 38 wavelet with 10 decomposition levels due to the broadband nature of the ionoacoustic signals. Hard thresholding technique was applied on the fifth level, with a threshold set to 10% of the maximum value of the detail coefficient at this level. The detail coefficients of the lower levels (corresponding to frequencies higher than 10 MHz) were set to zero.

In order to assess the evolution of the signal amplitude and phase shift with the proton pulse period including statistics, the 3000 acquisitions of each dataset were subdivided into 30 measurements (each measurement being the average of 100 acquisitions). The complete data analysis process is illustrated in Fig. S8 of the supplementary information. The amplitudes were obtained from the Fast Fourier transform (FFT) of the different signals, extracted at the temporal excitation frequency ($f_0$) which was determined from the trigger signals for each dataset. The analysis in the frequency domain was required to separate the high and low frequency components of the signal, particularly enhanced for the measurements in water. For the measurements without ripple filter, the FFT was calculated on a reduced time window (from ${21} \hbox {to} {22.5}{{\,\upmu \hbox {s}}}$) to not include the water tank entrance window signal. Therefore, zero-padding was performed in time domain to maintain an accurate frequency resolution $(df={5}{\,\hbox {kHz}})$. The signals were filtered using cascade-second order section and bandpass second order Butterworth filter (filter bandwidth set to ± 10% of $f_0$) prior the phase shift calculation. Thereafter, the phase was determined from the ratio of the two Hilbert transforms of the signals analyzed (CA and reference in water for a given period) and the phase shift was assessed from the mean value on a given time window (from ${21}\,\hbox {to}\,{22.5}{{\,\upmu \hbox {s}}}$ in agreement with the amplitude estimation).

### Simulation of the 22 MeV proton beam and optical photon dose depositions

The spatially resolved dose depositions inside the water tank and phantom were calculated using the FLUKA^32,33^ Monte Carlo code (Fluka2021.1beta, to be released at the beginning of 2021, using PRECISIOn defaults). The simulation geometry included the ${11.4}{\,\upmu \hbox {m}}$ thin Titanium beamline vacuum window and a 6cm air gap before entering the water tank through the first ${50}{\,\upmu \hbox {m}}$ polyimide foil (see supplementary Fig. S1). For the proton dose calculation, the content of the CA phantom was assumed to be water, with the same properties as the surrounding water (density of ${998}{\hbox {kg}\,\hbox {m}^{-3}}$ and ionization potential of 75eV), as validated in previous a study^34^.

For simulating the spread-out dose distribution, the ripple filter was additionally modelled in the FLUKA simulation geometry downstream of the Titanium window. A confocal microscope was used to perform one-dimensional profile scans of the filter surface perpendicular to the ripples (see supplementary Fig. S1). This served as the input to accurately model positions and thickness of aluminum (aluminum alloy ${\hbox {AlMg}_3}$) ridges and grooves in the simulation geometry. In order to account for experimental proton beam instability, the dose calculations with the ripple filter were performed with the proton beam hitting randomly six different areas of the aluminum structure (see supplementary Fig. S2).

Scintillation light production was activated in FLUKA to model the optical photon emission from water luminescence as proposed by Yabe et al.^19^ and Cherenkov light production was deactivated. The optical photons spectrum was defined using a dedicated user routine, randomly sampling the wavelength of the generated optical photons ($\lambda$) assuming a $\lambda ^{-2}$ distribution (see Fig. S9 in the supplementary information). The wavelength range of the generated and transported photons was limited to 300 nm to 700 nm, corresponding to the range for which water luminescence has been already experimentally observed^21^. The evolution of the absorption coefficient as a function of the wavelength was considered for both water and India ink, according to values reported in Pope et al.^35^ and Di Ninni et al.^22^, respectively, as depicted in Fig. S9 of the supporting information. For all the luminescence simulations, optical properties of the polyimide foil were assumed to be the same as water and the initial light yield was set to 0.1 photon/MeV, based on previously reported values^19^.

Doses were scored within a three dimensional Cartesian grid covering the entire range of the proton beam. Bin width was ${25}{\,\upmu \hbox {m}}$ axially and laterally.

### Pressure conversion and wave propagation from the 22 MeV proton beam

The simulated dose depositions (proton and optical photons) were used as input for the ionoacoustic wave propagation based on the open-source acoustic toolbox k-wave^36,37^ available for Matlab (MATLAB R2019a, MathWorks, Natick, MA). Considering the water temperature *T* of the water tank, the dose *D* given in $[\hbox {J}\,\hbox {kg}^{-1}]$ was converted on a voxel basis to the initial pressure $p_{0}$ [Pa] with ${\varvec{r}}$ being a vector denoting the spatial subscripts *x*,*y* and *z*:

1 $$\begin{aligned} p_{0}({\varvec{r}},T) = D({\varvec{r}}) * \varGamma ({\varvec{r}},T) * \rho ({\varvec{r}},T) \end{aligned}$$

whereas $\rho$ represents the density $[\hbox {kg} \,\hbox {m}^{-3}]$ and $\varGamma$ the dimensionless Grüneisen parameter $\varGamma =\frac{\beta c^2}{C_p}$, where $\beta$ is the medium compressibility, *c* is the speed-of-sound in that medium and $C_p$ is the isobaric specific heat capacity.

The simulation setup and parameters are summarized in Fig. S1 of the supplementary information. The medium properties (density, speed-of-sound and Grüneisen parameter) were varied along the beam axis in order to account for the different materials (air, polyimide and water). The India ink was assumed to have the same properties as water, estimated from temperature measurement during the experiments ($\rho ={998}[\hbox {kg} \,\hbox {m}^{-3}]$, $c_{water}={1484.1}\hbox {m}\,\hbox {s}^{-1}$ and $\varGamma =0.11$ at ${20.5}^{\circ }\hbox {C}$). The initial pressure was propagated using the k-space pseudospectral method, assuming instantaneous energy deposition (Dirac delta-spike proton pulse). The resulting pressure wavefront was integrated on a disk sensor positioned similarly to the experiments, herewith accounting for the spatial averaging of the acoustic wave due to the sensor geometry.

In all k-Wave simulations, an axial grid spacing of ${25}{{\,\upmu \hbox {m}}}$ was chosen to accurately model the ${50}{{\,\upmu \hbox {m}}}$ polyimide foil which is the source of high frequency oscillations in the ionoacoustic signal. A ${50}{{\,\upmu \hbox {m}}}$ lateral grid spacing was used to reduce the computational workload. The lateral grid spacing was chosen in the same order as the axial one to prevent any spurious oscillations of the proton dose in the frequency domain. The simulation time step was determined from the Courant-Friedrichs-Lewy (CFL) number which is given by the ratio of the distance traveled by the wave in one time step $\varDelta t$ to the grid spacing $\varDelta x$ ($CFL = \frac{c_{ref}\varDelta t}{\varDelta x}$), with $c_{ref}$ corresponding to the maximum speed-of-sound in the simulated geometry ($c_{ref}={2400}{\hbox {m}\,\hbox {s}^{-1}}$ in the polyimide foil). The CFL number was set to 0.3 as a good trade-off between numerical stability and simulation runtime^38^.

In order to account for proton beam instability during the experiments (lateral displacement), each setup e.g., ionoacoustic signals in water with ripple filter, was simulated for six different lateral positions of the proton beam. The pressure was then averaged considering a given weighting factor for each of the six simulated pressures. The individual weighting factors were determined by matching the lateral dimension of the total simulated dose distal to the titanium foil (average of the doses obtained at the different positions) to the EBT3 film measurement. The detailed comparison is shown in Fig. S2 of the supplementary information.

The simulated pressure wave was afterwards convolved with 1 or 25-cycle square pulse train of 400 ns period to mimic realistic non-delta like proton excitation^39^, in agreement with the experiments. The CMUT electrical impulse response was also taken into account, by filtering the simulated pressures using cascade-second order section and bandpass second order Butterworth filter (0.15–15 MHz). The filter parameters were defined in order to obtain good agreement between simulations and experiments.

### Photo-enhanced ionoacoustic simulations at clinical energy

The proton and optical doses were obtained from the FLUKA Monte Carlo code (version Fluka2021.1beta, using HADROTHErapy defaults). The simulation geometry was similar to the one previously reported by Würl et al.^40^ which modelled the beamline of the Rinecker Proton Therapy Center (Munich, Germany) for a 130 MeV proton beam, irradiating a water-equivalent phantom. The medium densities were chosen to correspond with the irradiation of a 2cm hepatic tumor surrounded by liver tissue. The tumor was positioned in order to have the Bragg peak inside the targeted volume and moved axially by 5 mm so that the Bragg peak scans the tumor volume (distance of the Bragg peak to the tumor entrance edge ranging from 4.64 mm to 19.44 mm, see setup illustrated in supplementary Fig. S11). The optical photon dose was simulated similarly as for the 22 MeV proton beam. The same optical photon absorption coefficient as in India ink was used for the hepatic tumor and the absorption in liver tissue was similar to the one in water.

All the medium properties (density, speed-of-sound and Grüneisen parameter) were determined on the basis of the compute tomography Hounsfield units (HU) reported by Berber et al.^41^ (34 HU and 49 HU for the tumor and liver, respectively), using the empirical conversion table proposed by Yu et al.^42^. In order to reduce the computation workload imposed by the fine axial binning required to propagate the high frequency photoacoustic pressure ($\varDelta x={50}{{\,\upmu \hbox {m}}}$ and CFL=0.3), simulations were performed in 2D and the pressure wave was recorded using an ideal point sensor positioned at 27 cm from the phantom entrance. As done previously, the ionoacoustic and photoacoustic simulations were performed separately and the two contributions were summed afterward, applying a correction factor on the photoacoustic signal (${5\times 10^{5}}$ or ${1\times 10^{6}}$). The signals were thereafter convolved with the proton pulse time profile.

A ${2.5}{{\,\upmu \hbox {s}}}$ Gaussian proton pulse was used to model ionoacoustic signals from synchro-cyclotrons-based clinical facilities with a charge per pulse set to 0.16pC (${1\times 10^{6}}$ protons/pulse), corresponding to realistic treatment conditions. The temporal micro-structure observed from synchrotrons was assessed on the basis of the values reported by Martins et al.^43^, with a special effort to model the stochastic nature of the synchrotron micro-structure. Individual micro-pulses were randomly sampled following a Gaussian distribution $(\sigma =19.75\,\hbox {ns}$). The number of samples was chosen to correspond with clinically relevant fluence of $3.2\times 10^{9}$ protons/second. The micro-pulse amplitude was scaled using a Poisson distribution (mean value set to 1) to account for fluence fluctuation. The process was repeated in order to obtain a 50 ms burst, with individual micro-pulses repeated each 228 ns (pulse repetition frequency of 4.39 MHz). The micro-pulse structure obtained is illustrated in Fig. S11 of the supporting information and found in good agreement with data reported by Martins et al.^43^ .

The beats and distance from the Bragg peak to the tumor edge were determined from the Fourier transform of the filtered signal spectra. The frequency sprectra of the ionoacoustic signals were determined from a first Fourier transform (from time to frequency domain, depicted in Fig. 6e). Thereafter, the absolute values of the signal spectra were filtered (cascade-second order section and lowpass second order Butterworth filter) in order to obtain their envelopes. The cutoff time for the filtering was decided based on the signal time-of-flight (set to ${120}{{\,\upmu \hbox {s}}}$). Finally, the filtered spectra were Fourier transformed (from frequency to time domain) and the distance was determined from the speed-of-sound in the tumor $({1556.9}{\hbox {m}\,\hbox {s}^{-1}})$.

## Supplementary Information

Supplementary material 1
