# Supplementary material for: Enhancement of the ionoacoustic effect through ultrasound and photoacoustic contrast agents
Source: Sci Rep. 2021 Feb 1;11:2725. doi: 10.1038/s41598-021-81964-4 (PMC7851171; doi:10.1038/s41598-021-81964-4)
Supplement: Supplementary file 1 — Supplementary material 1 [file 41598_2021_81964_MOESM1_ESM.pdf]

Supplementary information: Enhancement of the ionoacoustic effect through ultrasound and photoacoustic contrast agents

Julie Lascaud, Pratik Dash, Matthias Würl, Hans-Peter Wieser, Benjamin Wollant, Ronaldo Kalunga, Walter Assmann, Dirk-André Clevert, Alfredo Ferrari, Paola Sala, Alessandro Stuart Savoia and Katia Parodi

Corresponding authors: J.Lascaud@physik.uni-muenchen.de
Katia.Parodi@physik.uni-muenchen.de

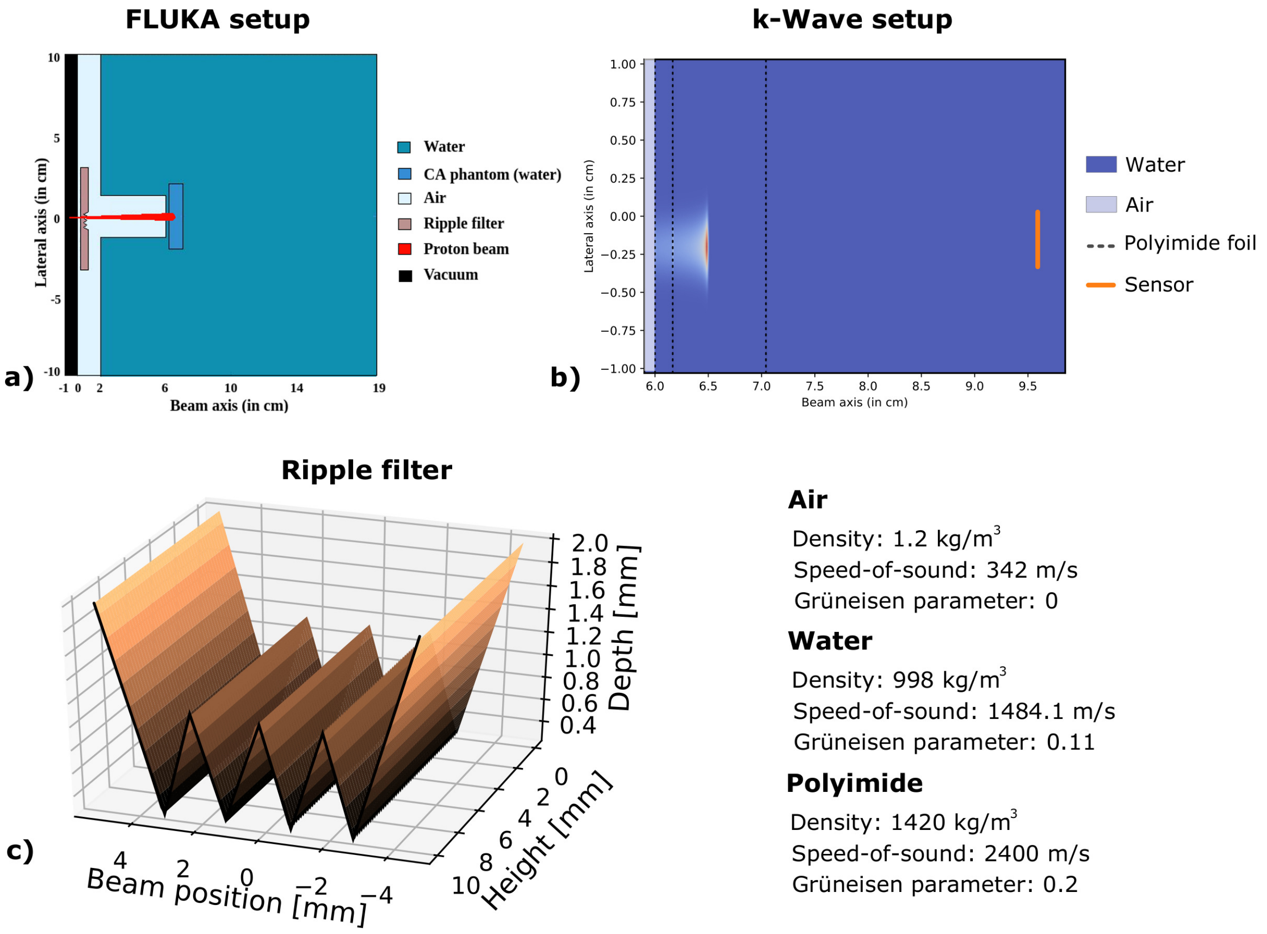

Figure S1: Simulation setups. (a) Schematic top view of the geometry used in the FLUKA code to simulate the 22 MeV proton beam. (b) Schematic top view of the geometry used in k-Wave to model the propagation of the ionoacoustic pressures. (c) 3D representation of the ripple filter determined by confocal microscopy.

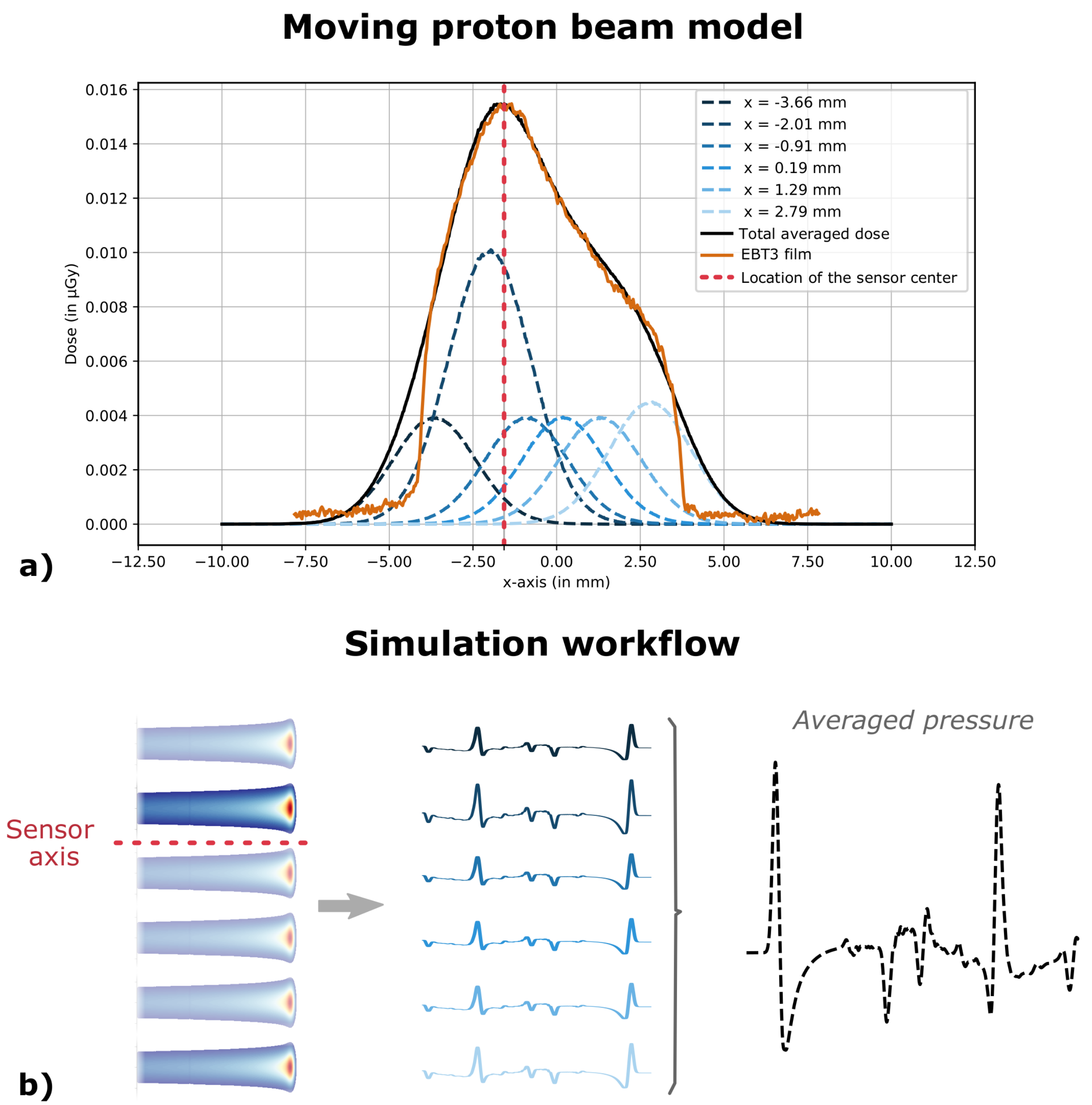

Figure S2: Moving proton beam model. (a) Comparison of the EBT3 film measurement (orange) and simulated total dose (black) both located distal to the Titanium foil. The blue dashed lines show the six weighted pencil beams. For the k-Wave simulations, the sensor was laterally positioned at the maximum of the total dose (red dash line). (b) Schematic representation of the simulation workflow of ionoacoustic signal production. Weighted pressures were simulated from each individual pencil beam and summed.

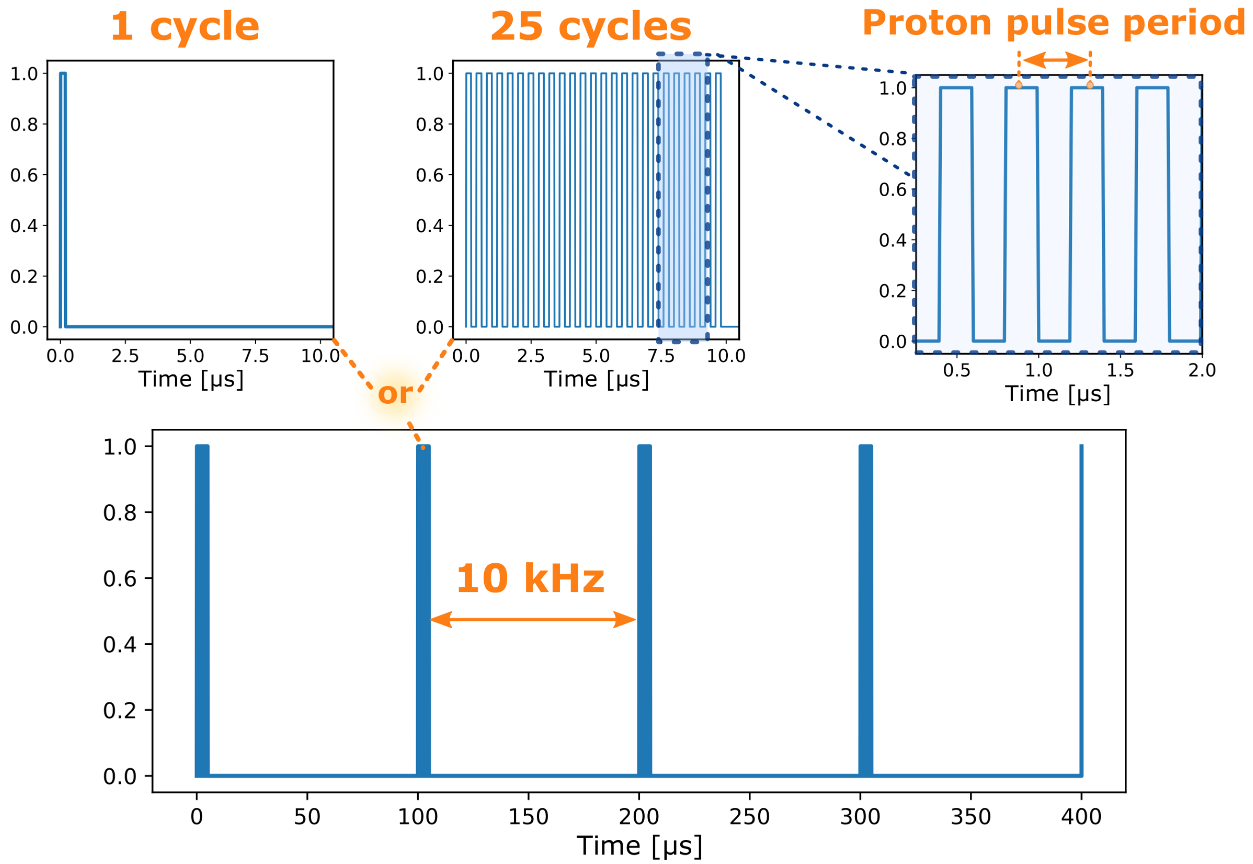

Figure S3: Schematic representation of the proton pulse time profile as controlled by the function generator. For all the measurements, proton pulse emissions were repeated each 100 µs (10 kHz repetition rate). The single proton pulse measurements were done using 1-cycle square pulses of 200 ns, whereas 25-cycle bursts of various proton pulse period (from 350 ns to 660 ns) and a 50 % duty cycle (time on = time off) were used to investigated the microbubble resonance.

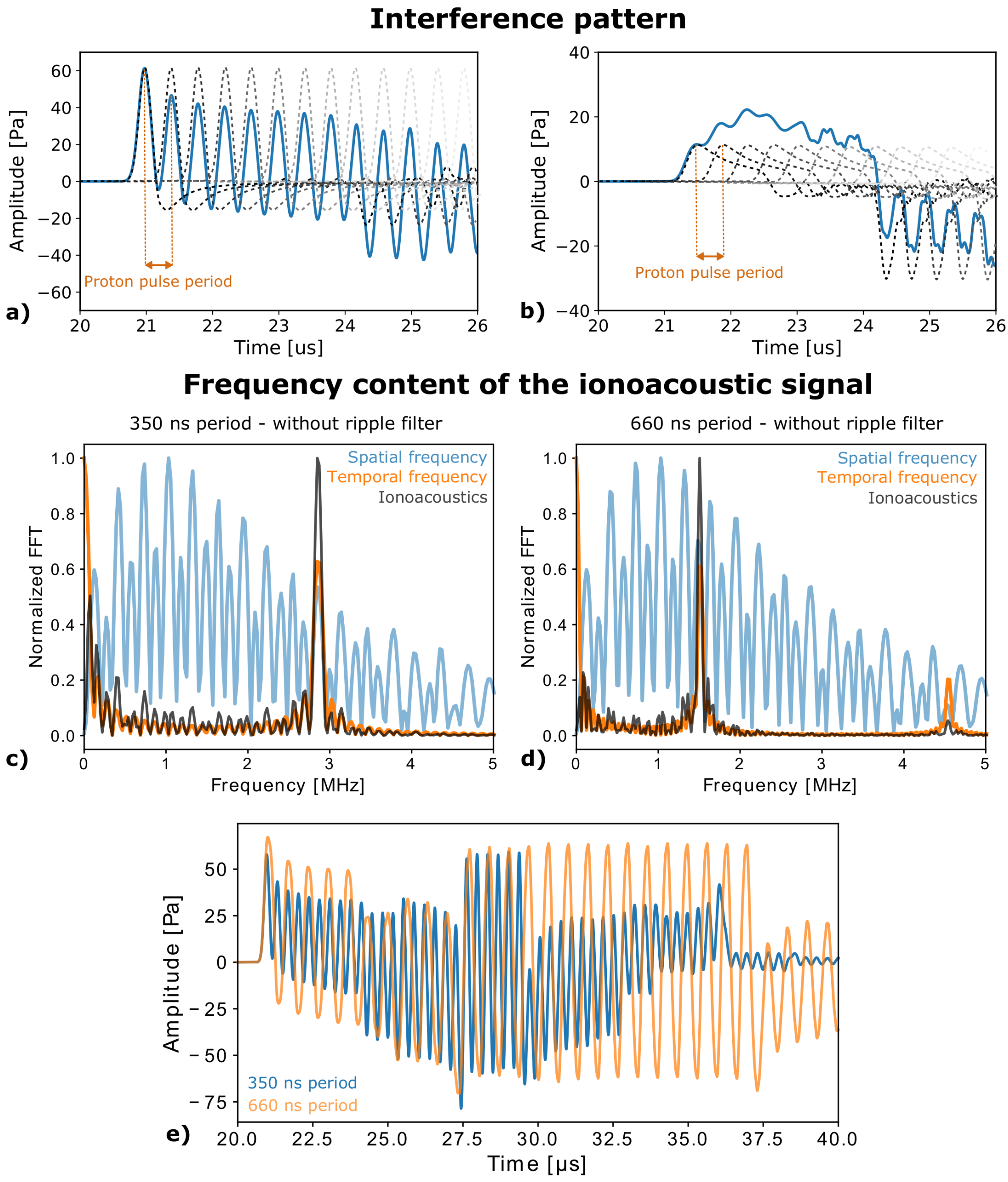

Figure S4: Illustration of the inteferences occurring with 25-cycle proton pulse burst (a) for the mono-energetic beam and (b) spread-out Bragg peak. The resulting ionoacoustic signal (blue) is the superposition of individual ionoacoustic signals (gray dashed lines) separated in time by the proton pulse period. Frequency spectra of the simulated ionoacoustic signals without ripple filter for 25-cycle burst with a period of (c) 350 ns and (d) 660 ns. Spatial frequency (blue) deduced from the simulated pressure before convolution with the proton pulse time profile, FFT of the ionoacoustic signal (black) after convolution with the proton pulse time profile and FFT of the proton pulse time profile (orange). (e) Simulated ionoacoustic signals for the mono-energetic beam and a 25-cycle burst with a period of 350 ns (blue) and 660 ns (orange).

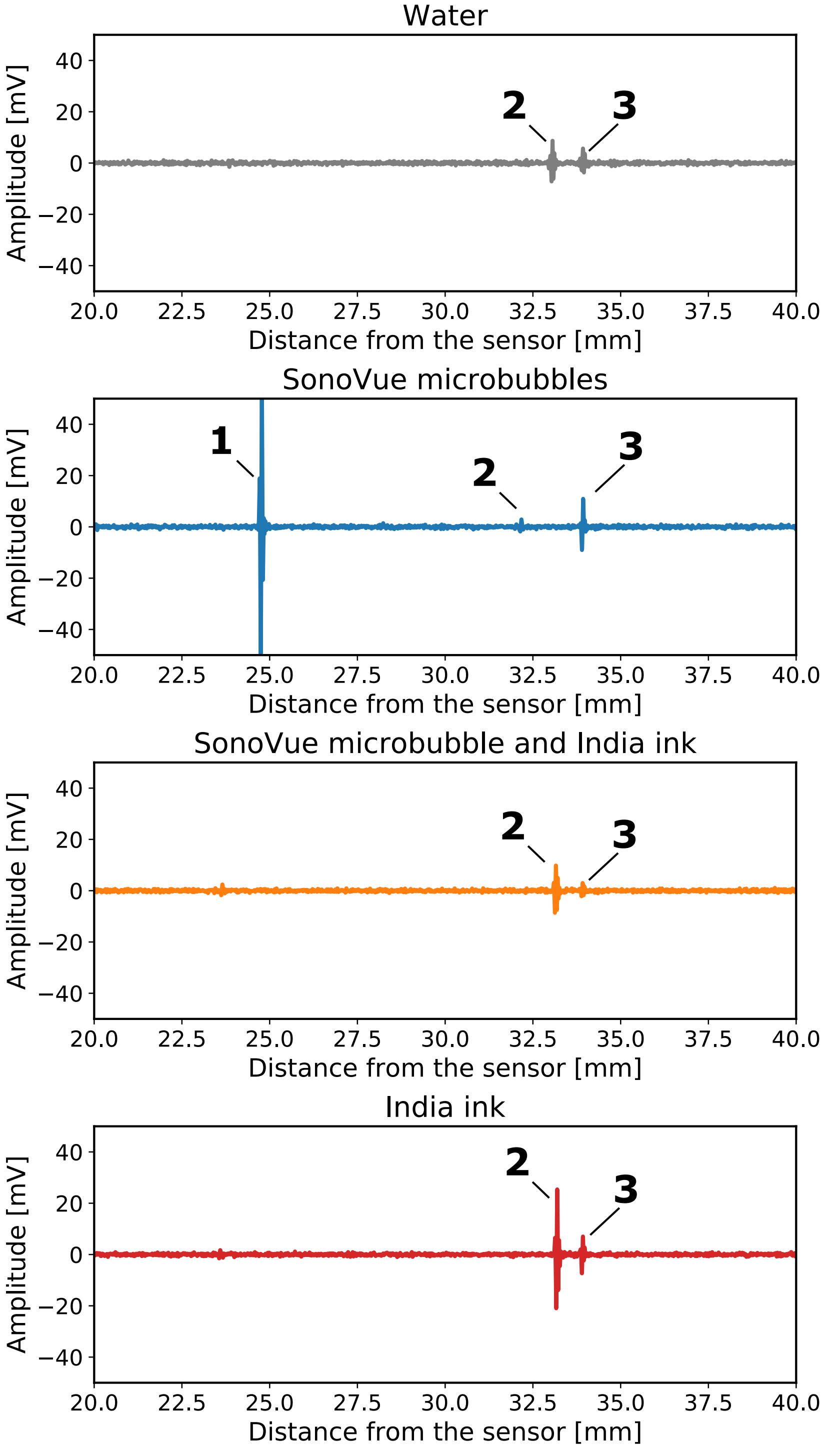

Figure S5: Ultrasound pulse-echoes depicting (1) the polyimide foil distal to the Bragg peak, (2) the polyimide foil at the CA entrance and (3) the entrance window of the water tank. CA phantom filled with: water (black), SonoVue micobubbles (blue), SonoVue microbubbles and India ink (orange) and India ink diluted in water (red). The x-axis shows the distance relative to the sensor assuming a constant speed-of-sound of $1484.1\,\mathrm{m\,s^{-1}}$.

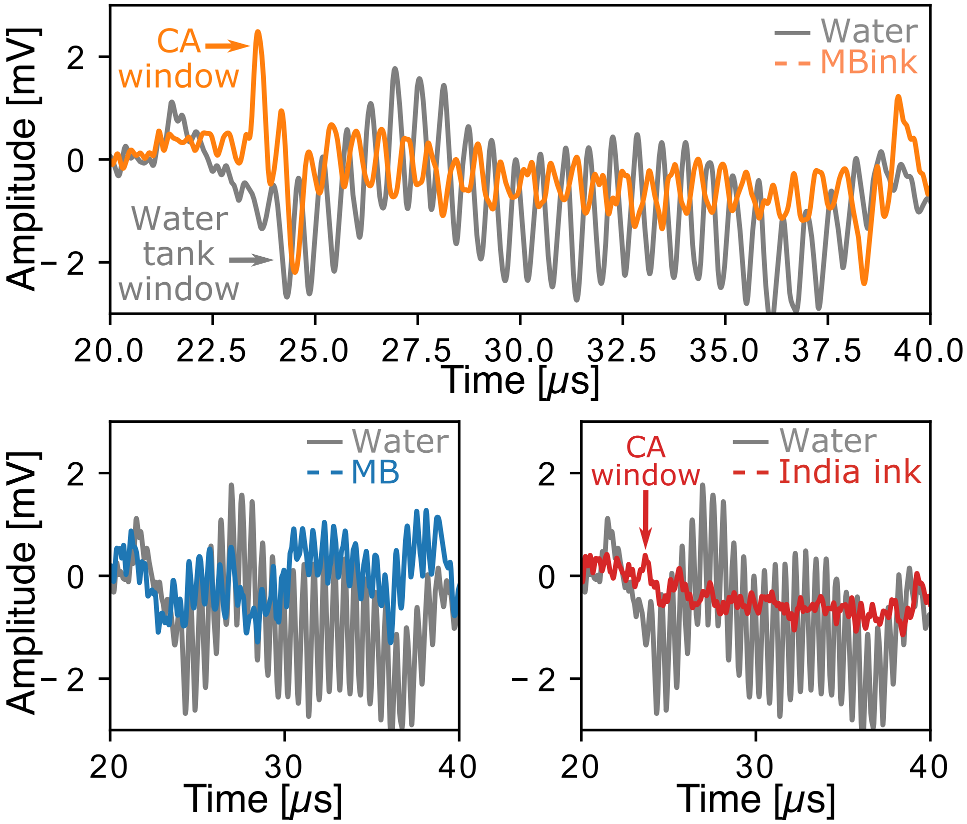

Figure S6: Ionoacoustic measurements with ripple filter (25 cycles, pulse period = 590 ns), in water (gray), with microbubbles combined with India ink (orange), with microbubbles (blue) and India ink alone (red). For the India ink-based CA, the destructive interference between the ionoacoustic signal generated at the CA window and the signal emitting from the water tank entrance window leads to a drop of amplitude after 24.3 μs, not observed for the measurements in water or with microbubbles. The interferences reveal that the CA window signal is in the same order of magnitude as the water tank entrance signal. Since the interferences are only observed for India ink-based CA, they suggest an underlying additional photoacoustic effect enhanced due to the higher optical absorption in the CA.

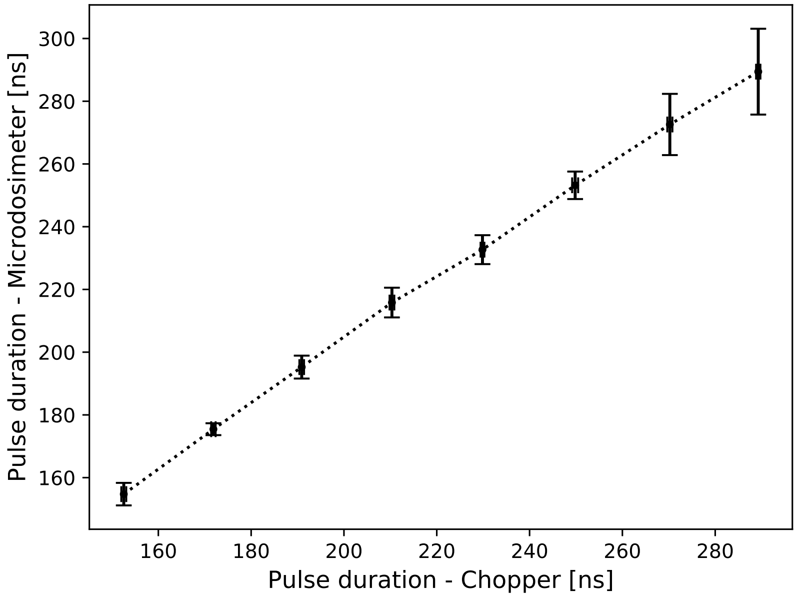

Figure S7: Evolution of the proton pulse duration measured with the fast silicon detector as a function of the pulse duration obtained from the accelerator chopper system. All error bars correspond to measurement uncertainties obtained from 50 consecutive measurements and given for a confidence level of 99 %.

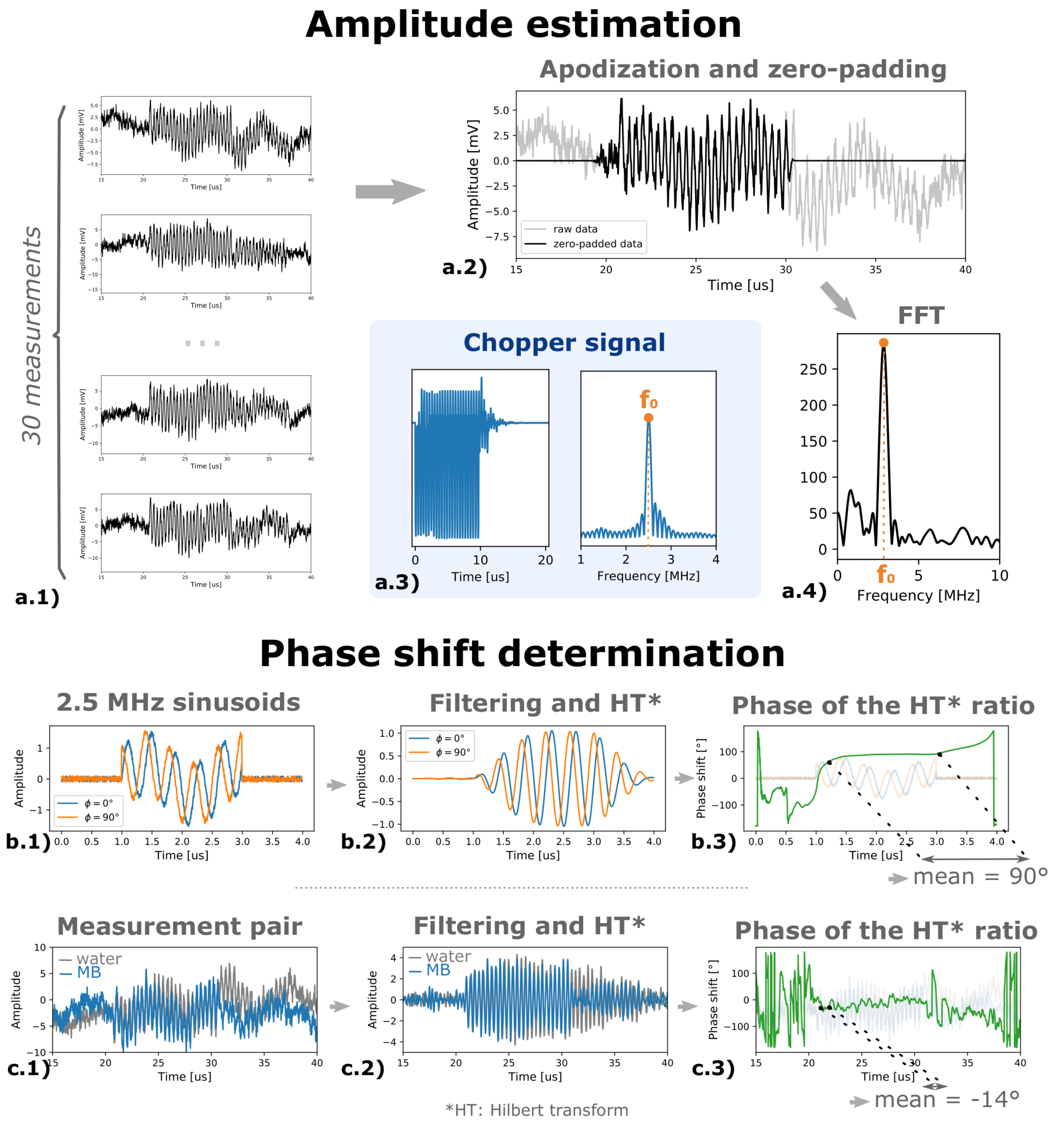

Figure S8: Data post-processing. (a.1) Each dataset was subdivided into 30 measurements. (a.2) Apodization and zero-padding were used in order to analyze the signals on a reduced time window. (a.3) The temporal excitation frequency (f_0) was determined from the trigger signal FFT for each dataset and (a.4) the signal amplitude was extracted from the FFT of the zero-padded signals at f_0. Example of phase shift determination (b.1) for 2.5 MHz sinusoid with a 90° phase shift. (b.2) Hilbert transform (HT) of the 2.5 MHz filtered signals and (b.3) phase estimation from the ratio of the two HT. Example of experimental phase shift determination. (c.1) 25-cycle and spread-out Bragg peak in water (gray) and with microbubbles (blue). (c.2) HT of the signals after bandpass filtering (± 10 % of f_0). (c.3) Phase estimation on the same time window as for the amplitude evaluation.

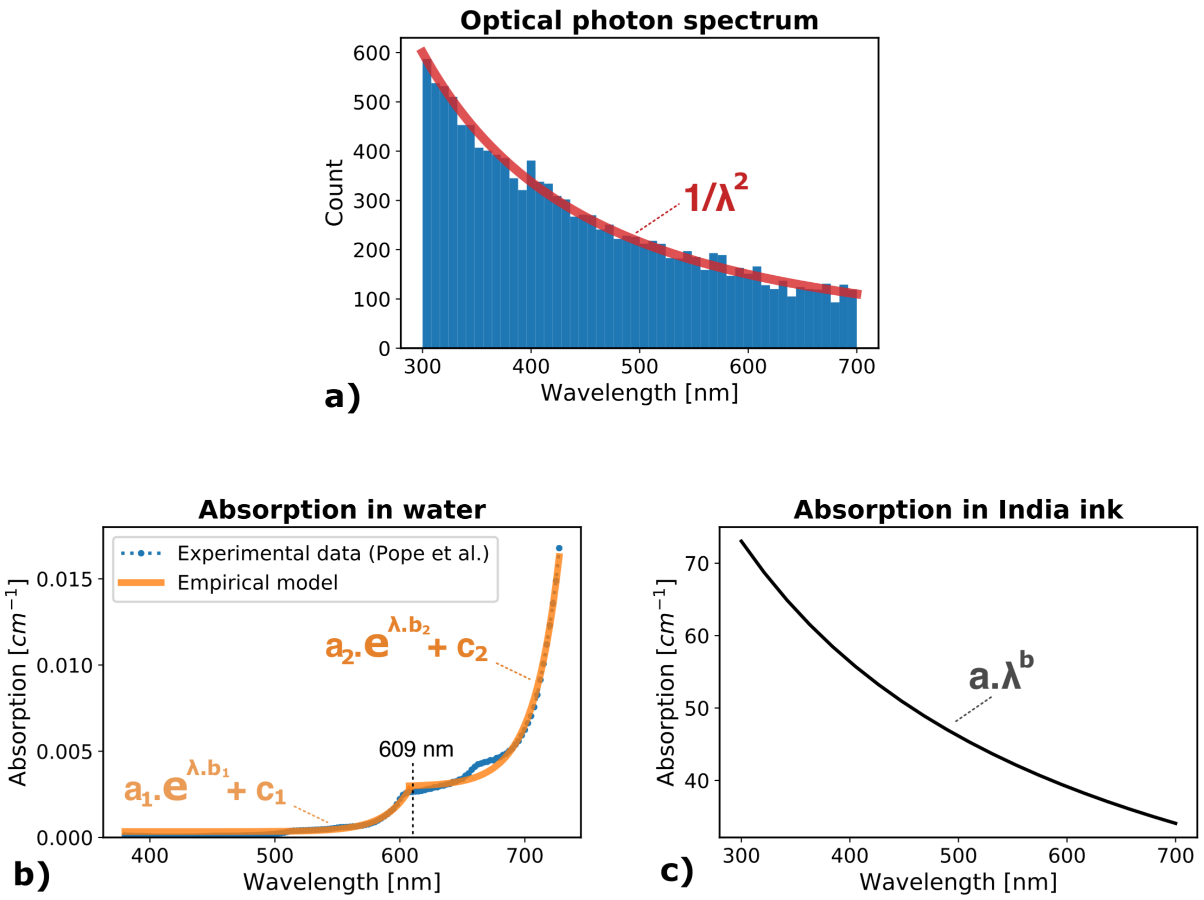

Figure S9: Luminescence simulation parameters. (a) Optical photon spectrum. (b) Absorption coefficient [in cm^{-1}] in water as a function of the wavelength (λ) [in cm] approximated from Pope *et al.*, with $a_1 = 2.78 \times 10^{-16}$, $b_1 = 4.91 \times 10^7$ and $c_1 = 3.40 \times 10^{-4}$ for $\lambda \preceq 609\,\text{nm}$. Otherwise, $a_2 = 9.95 \times 10^{-18}$, $b_2 = 4.79 \times 10^7$ and $c_2 = 2.95 \times 10^{-3}$. (c) Absorption coefficient [in cm^{-1}] in India ink as a function of (λ) [in cm] approximated from Di Ninni *et al.*, with $a = 9.84 \times 10^{-5}$ and $b = -0.9$.

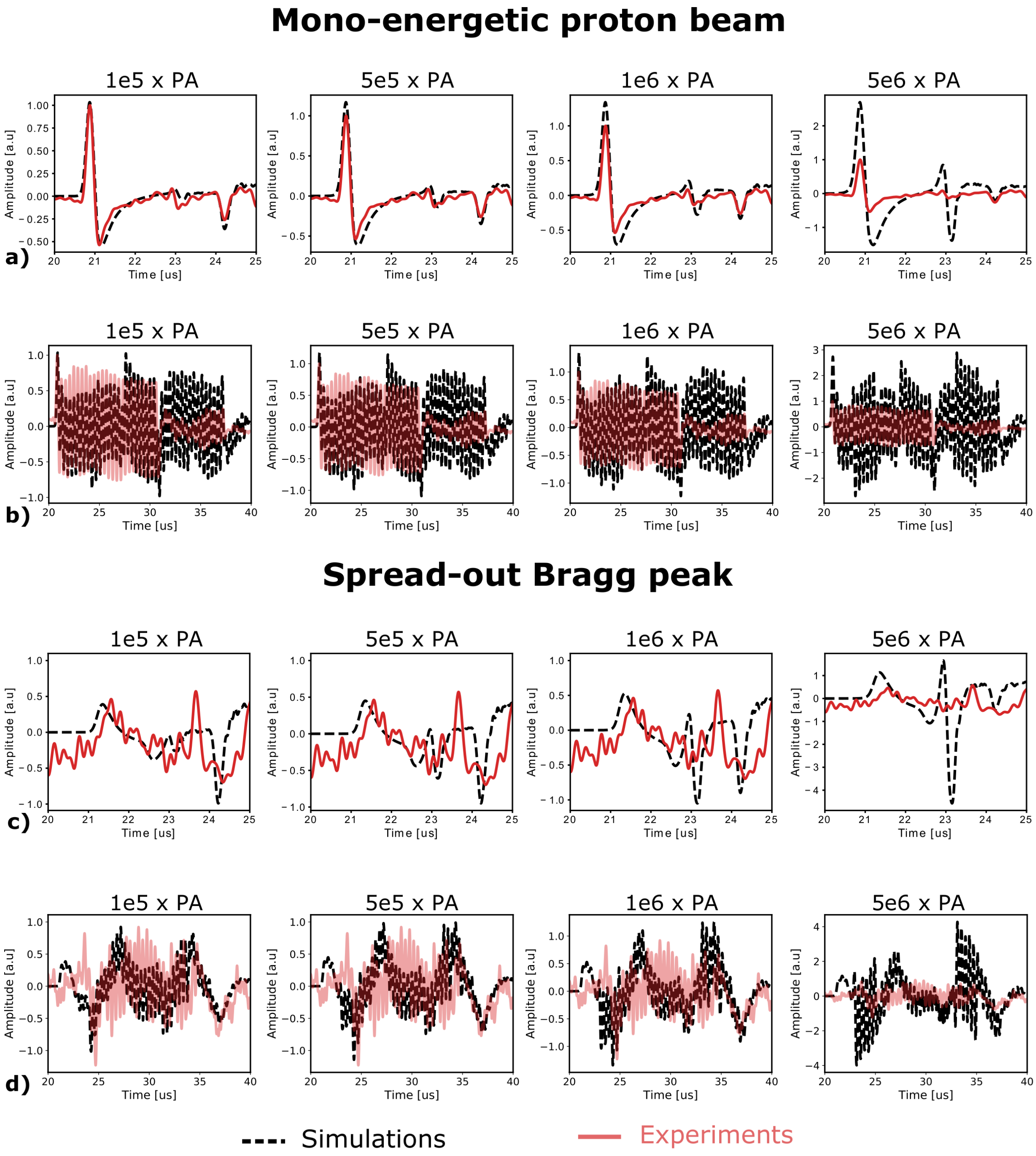

Figure S10: Luminescence simulations from the 22 MeV proton beam in India ink. Comparison between the experiments (red) and simulation results (black dashed line) for the mono-energetic proton beam using (a) single-proton pulse or (b) 25-cycle burst and ionoacoustic pressures from the spread-out Bragg peak with (c) single-proton pulse or (d) 25-cycle burst. Each dataset is shown for different correction factors of the photoacoustic emission (PA), ranging from 1×10^5 to 5×10^6 and equivalent to a light yield from 1×10^4 photons/MeV to 5×10^5 photons/MeV, respectively.

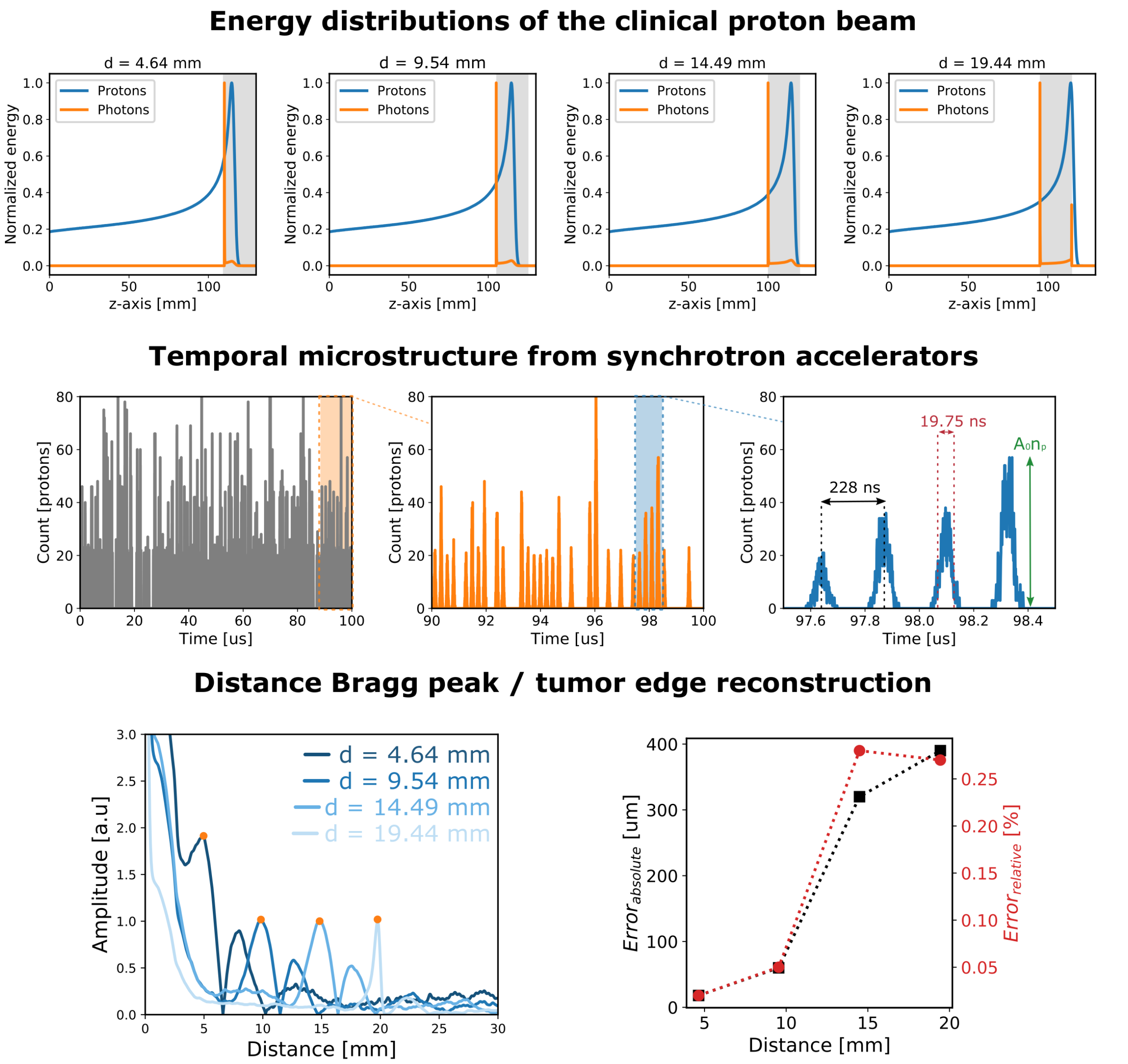

Figure S11: Simulated photo-enhanced ionoacoustics from a clinical proton beam. (a) Energy distributions of the proton beam (blue) and optical photon absorption (orange) obtained moving the tumor along the proton beam axis. The gray area represents the position of the 2 cm-thick tumor. (b) Representation with different time scales of the temporal micro-structure of proton beams from synchrotrons. The initial amplitude A_0 of each micro-pulse (fluence of 3.2×10^9 protons/s) is randomly scaled by a n_p factor modelling fluence fluctuation, where n_p follows a Poisson distribution of mean value equal to 1. (c) Reconstruction of the distance signal beats depending on the distance from the Bragg peak to the tumor entrance edge. (d) Absolute error on the distance estimation (black) and error relative to the range (red). Note that, the density of the tumor and liver tissue being different, the proton beam range varies depending on the tumor position.
